# New Insights Into Mitochondrial DNA Reconstruction and Variant Detection in Ancient Samples

**DOI:** 10.3389/fgene.2021.619950

**Published:** 2021-02-18

**Authors:** Maria Angela Diroma, Alessandra Modi, Martina Lari, Luca Sineo, David Caramelli, Stefania Vai

**Affiliations:** ^1^Dipartimento di Biologia, Università degli Studi di Firenze, Florence, Italy; ^2^Dipartimento di Scienze e Tecnologie Biologiche, Chimiche e Farmaceutiche, Università degli Studi di Palermo, Palermo, Italy

**Keywords:** ancient DNA, mitochondrial DNA, NUMTs, heteroplasmy, variant detection

## Abstract

Ancient DNA (aDNA) studies are frequently focused on the analysis of the mitochondrial DNA (mtDNA), which is much more abundant than the nuclear genome, hence can be better retrieved from ancient remains. However, postmortem DNA damage and contamination make the data analysis difficult because of DNA fragmentation and nucleotide alterations. In this regard, the assessment of the heteroplasmic fraction in ancient mtDNA has always been considered an unachievable goal due to the complexity in distinguishing true endogenous variants from artifacts. We implemented and applied a computational pipeline for mtDNA analysis to a dataset of 30 ancient human samples from an Iron Age necropolis in Polizzello (Sicily, Italy). The pipeline includes several modules from well-established tools for aDNA analysis and a recently released variant caller, which was specifically conceived for mtDNA, applied for the first time to aDNA data. Through a fine-tuned filtering on variant allele sequencing features, we were able to accurately reconstruct nearly complete (>88%) mtDNA genome for almost all the analyzed samples (27 out of 30), depending on the degree of preservation and the sequencing throughput, and to get a reliable set of variants allowing haplogroup prediction. Additionally, we provide guidelines to deal with possible artifact sources, including nuclear mitochondrial sequence (NumtS) contamination, an often-neglected issue in ancient mtDNA surveys. Potential heteroplasmy levels were also estimated, although most variants were likely homoplasmic, and validated by data simulations, proving that new sequencing technologies and software are sensitive enough to detect partially mutated sites in ancient genomes and discriminate true variants from artifacts. A thorough functional annotation of detected and filtered mtDNA variants was also performed for a comprehensive evaluation of these ancient samples.

## Introduction

Genetic material recovered from ancient samples has particular characteristics due to degradations that occurred through time. Endogenous ancient DNA (aDNA) molecules are often retrieved in low copy number, with the co-presence of possible exogenous contaminant DNA, and are characterized by high fragmentation and a typical pattern of damage at read termini. Degradation of the genetic material is due to factors such as temperature, pH, and processes as hydrolysis and oxidation that act on the biological sample through time. Fragmentation and decline of the amount of endogenous molecules are mainly due to depurination caused by hydrolysis while deamination of cytosines tends to occur at the 5′ ends, being translated in misincorporations C to T (C > T) at 5′ and G to A (G > A) at 3′ in the final sequence obtained from a double-stranded library (Briggs et al., 2007). In many studies on ancient samples, mitochondrial DNA (mtDNA) has been preferred as a target because of its high number of copies in each cell and consequently its higher availability with respect to nuclear DNA. It represents also a useful marker for evolutionary and population genetics analysis extensively applied on ancient samples (Modi et al., 2017; Chyleński et al., 2019; Ehler et al., 2019; Modi et al., 2019, 2020a,b; Översti et al., 2019; Vai et al., 2019; Juras et al., 2020; Yang et al., 2020). However, besides contamination by exogenous DNA samples, another source of artifacts generally affecting mtDNA analysis is represented by nuclear sequences of mitochondrial origin (NumtS), barely explored in aDNA (den Tex et al., 2010; Samaniego Castruita et al., 2015).

Next-generation sequencing (NGS) technologies have revolutionized the field of aDNA. They allow: (i) recovering short molecules, even <50 bp long, that can represent a considerable amount of the endogenous fraction in archeological samples (Prüfer et al., 2010; Dabney et al., 2013); (ii) direct sequencing with no targeted amplification steps; and (iii) generating high amounts of sequence data at low cost. Unlike traditional sequencing methods, NGS also allows detecting misincorporation patterns, useful for differentiating between endogenous aDNA and present-day contaminants (Briggs et al., 2007; Sawyer et al., 2012), thus the analysis of damage pattern started to be essential for the authentication of the results obtained from ancient samples and for managing potential contamination. In addition to specific experimental strategies, optimized for the analysis of ultrashort DNA fragments (Rohland and Hofreiter, 2007; Dabney et al., 2013) and for library preparation and target enrichment on degraded samples (Maricic et al., 2010; Meyer and Kircher, 2010; Gansauge and Meyer, 2013; Rohland et al., 2015), dedicated bioinformatics pipelines were developed to improve the analysis of ancient molecules (Kircher, 2012; Shapiro and Hofreiter, 2012; Schubert et al., 2014; Peltzer et al., 2016). Furthermore, specific tools for the analysis and handling of molecule degradation were developed. For instance, mapDamage (Ginolhac et al., 2011), mapDamage2.0 (RRID:SCR_001240) (Jónsson et al., 2013), and DamageProfiler (Neukamm et al., 2020) packages allow to describe the misincorporation and length patterns. PMDtools (Skoglund et al., 2014) compute damage patterns and identify degraded sequences, allowing to decontaminate ancient genomes. Contamination estimates for human mtDNA are provided by tools such as contamMix (Fu et al., 2013), ContamLD (Nakatsuka et al., 2020), AuthentiCT (Peyrégne and Peter, 2020), and schmutzi (Renaud et al., 2015), the latter providing the reconstruction of the endogenous consensus sequence.

However, with rapid advancements in NGS technologies, bioinformatics pipelines are required to constantly evolve and/or be updated, promising enhancements in data analysis. Although the main steps of a workflow are well established, novelties in algorithms and approaches are generally introduced, often significantly affecting results. One of these steps is variant calling, which should consider the peculiarities of its target, such as ploidy and types of tissue (germinal or somatic), and be customized according to the given input. A very common approach for variant calling in aDNA is to use “pseudo-haploid” data due to their low coverage (Korneliussen et al., 2014; Barlow et al., 2020), where at each known polymorphic site, one sequencing read is randomly picked in order to represent a haploid genotype of that individual, avoiding to introduce bias if the reads were a random representation of the chromosomes carried by the individual but still affected by a reference bias, which can lead to loss of heterozygosity (Günther and Nettelblad, 2019). Variant calling in mtDNA deserves a separate mention because of the peculiarities of mitochondrial polyplasmic genetics and the possibility of the coexistence of different mtDNA genotypes within the same cell, tissue, or individual, a condition known as heteroplasmy. Resolving and reporting heteroplasmy are important considerations because it improves the consensus quality and downstream analyses (Rathbun et al., 2017). With this in mind, specific pipelines for mtDNA analysis in modern DNA samples have been generated (Guo et al., 2013; Calabrese et al., 2014; Vellarikkal et al., 2015; Weissensteiner et al., 2016a; Ishiya and Ueda, 2017; Rueda and Torkamani, 2017) and a variant caller implemented in the GATK package (RRID:SCR_001876) (McKenna et al., 2010), namely, Mutect2 (Benjamin et al., 2019), initially developed for somatic mutation detection in tumor samples, has been recently adapted to call mtDNA variants. A number of parameters are settable in Mutect2, including clipping of artifacts associated with end repair insertions near inverted tandem repeats, prevalent when DNA is damaged. Moreover, several filters can be applied to Mutect2 calls using a specific function provided in the package, making its application to aDNA possible. A Variant Call Format (VCF) file is provided, featuring many sequencing information, including the allele fraction (AF) for each variant position. To the best of our knowledge, no previous study used Mutect2 for ancient mtDNA analysis as well as no survey was thoroughly performed on potential heteroplasmy in ancient samples, although some useful suggestions for discriminating true variants from artifacts were provided in the forensic field, where DNA samples are frequently poorly preserved and damaged (Rathbun et al., 2017). In this work, we evaluated the efficacy of Mutect2 for variant calling in aDNA by introducing its usage in a pipeline we developed and applied to real and simulated datasets. To this aim, prevailing alleles were identified, used to assemble mtDNA consensus sequences, and compared with those detected by a well-used tool in the aDNA field, schmutzi (Renaud et al., 2015). An accurate functional annotation of all the variants obtained after a stringent filtering was performed to further evaluate variants and potential heteroplasmy levels, providing novel insights on aDNA.

## Materials and Methods

### Samples

To test the efficiency of our computational pipeline for mtDNA reconstruction and variant detection, we analyzed 30 Iron Age samples from Polizzello (Sicily, Italy), an archeological site in the heart of Sikania dated at IX–VII centuries BC (Messina et al., 2008). Molecular analysis was carried out in the Laboratory of Anthropology and Paleogenetics at University of Florence on petrous bones, where endogenous aDNA is better preserved than in other anatomical districts (Gamba et al., 2014; Pinhasi et al., 2015), following strict guidelines to prevent modern contamination during all experimental steps (Llamas et al., 2017). After decontamination of the outer part of the bone by brushing and UV irradiation, the bone powder was sampled using a microdrill and disk saw set at low speed to avoid heating, selecting the densest area of inner ear part of the petrous bone (Pinhasi et al., 2015). For each sample, DNA was extracted from 50–70 mg of powder as described by Dabney et al. (2013). After library preparation following a custom double-indexing protocol optimized for ancient samples (Meyer and Kircher, 2010; Kircher et al., 2012), mtDNA molecules were selected using hybrid capture target enrichment strategy (Maricic et al., 2010). Enriched libraries were then sequenced on Illumina NovaSeq 6000 platform: 14 samples were sequenced in single-end mode (1 × 100 + 8 + 8) and 16 in paired-end (2 × 100 + 8 + 8) (Supplementary Table S1).

### Data Preprocessing

**FIGURE 1:**
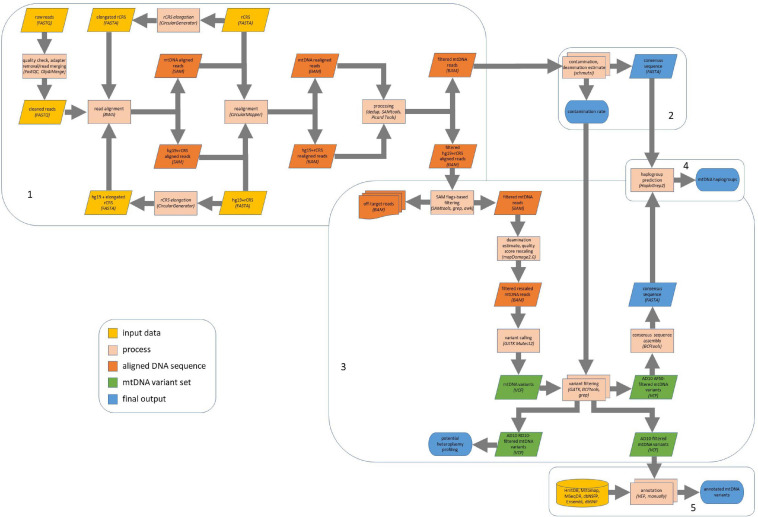
Computational pipeline for ancient mitochondrial DNA (mtDNA) analysis. Our computational pipeline comprises five main steps: **(1)** read alignment and preprocessing and postprocessing; **(2)** contamination analysis by schmutzi and consensus sequence assembly; **(3)** variant calling by GATK Mutect2, variant filtering, and consensus sequence assembly; **(4)** haplogroup prediction; **(5)** variant annotation. The alignment required revised Cambridge Reference Sequence (rCRS) as reference sequence to get a suitable input to schmutzi, while we used mtDNA reads aligned onto the whole genome (hg19 with rCRS as a mitochondrial reference sequence) for variant calling. AD10, minimum variant allele depth = 10; AF50, minimum allele fraction = 50%; RD10, minimum reference allele depth = 10.

The complete bioinformatics pipeline is shown in Figure 1. All the guidelines for command line tools used are provided as Supplementary Data. After quality check by FastQC^1^ (RRID:SCR_014583, v0.11.7), paired-end sequencing data in FASTQ format were first merged using *Clip&Merge* function (v1.7.6) from EAGER software (v1.92.37) (Peltzer et al., 2016), which also allowed to remove adapters from both paired- and single-end reads. Sequences with read length < 30 were discarded (–l 30), minimum base quality for quality trimming was set to 30 (–q 30). Sequences were clipped also when one nucleotide aligned with adapters (–m 1).

### Contamination Analysis and Mitochondrial DNA Consensus Calling

Read alignment was performed by BWA (RRID:SCR_010910, v0.7.17) *aln*, followed by *samse* (for single-end reads) or *sampe* (for paired-end reads) functions (Li and Durbin, 2009), onto an elongated version of the mitochondrial genome, obtained by adding the first 500 bases to the end of the revised Cambridge Reference Sequence (rCRS, GenBank: J01415.2) using the *CircularGenerator* function from EAGER. To increase the mapping efficiency of ancient sequences, seeding was disabled (–l 16,500) and the fraction of missing alignments, given 2% uniform base error, was set to 0.01 (Schubert et al., 2012). Then, reads were realigned onto the unmodified reference sequence by the *CircularMapper* method implemented in EAGER in order to improve read mapping to a circular sequence. SAMtools (RRID:SCR_002105, v1.3.1) *view*, *sort*, and *index* functions (Li H. et al., 2009) and Picard Tools (RRID:SCR_006525) *CleanSam^2^* (v2.17.3) were used for post-alignment processing, and duplicate removal was performed by *dedup* method (v0.12.1) from EAGER. Reads with mapping quality (MQ) ≥ 30 were selected, and their MD field was fixed using SAMtools *calmd*. We then used schmutzi (Renaud et al., 2015) to estimate the contamination and reconstruct the endogenous mitochondrial consensus sequence for each sample by exploiting deamination patterns and a database of putative contaminants (197 human mitogenomes deriving from a worldwide sampling) provided by the software package. Since samples were generated using non-UDG-treated libraries, 12 bases were considered to be deaminated on each read end (–lengthDeam 12). Contamination levels with likelihood values were reported for all samples in the database, allowing to identify the most likely contaminant sample.

### Variant Calling and Filtering

Raw reads were also aligned using BWA onto the human reference genome assembly hg19, including the elongated rCRS sequence, and then realigned onto the unmodified reference sequence by EAGER *CircularMapper*. The parameters in BWA and the post-alignment processing remained the same as described in section “*Materials and Methods*, *Contamination Analysis and Mitochondrial DNA Consensus Calling.”* Primary alignments to mtDNA were then selected from deduplicated Binary Sequence Alignment/Map (BAM) files by specifying “–F 4 –F 256 –F 2048” flagstats to samtools *view* command in order to remove unmapped, secondary, and supplementary alignments, respectively. Although a stringent MQ-based filtering (MQ ≥ 30) is generally sufficient to this aim, we used *grep* and *awk* commands in bash to filter out other reads with suboptimal hits found by BWA, by exploiting BAM tags XA and X1, mostly computed for single-end read mapping. In this fashion, we ensured to exclude off-target sequences, including nuclear DNA sequences and reads ambiguously mapped to both nuclear and mitochondrial genome, which could be potential NumtS sequences. We then applied mapDamage2.0 (RRID:SCR_001240, v2.0.8) (Jónsson et al., 2013) to mitochondrial reads in BAM format with MQ ≥ 30 in order to quantify DNA damage patterns in ancient sequences, enabling the “–rescale” parameter to downscale quality scores of likely damaged positions in the reads and obtain new BAM files. Variant calling in each single sample was performed by using GATK (RRID:SCR_001876, v4.1.4.1) *Mutect2* caller (McKenna et al., 2010; Benjamin et al., 2019), specifically designed for somatic variants, by setting the mitochondrial mode, which automatically sets parameters appropriately for calling on mitochondria (–mitochondria-mode –initial-tumor-lod 0 –tumor-lod-to-emit 0 –af-of-alleles-not-in-resource 4e-3 –pruning-lod-threshold -4). By default, Mutect2 identifies and clips artifacts generated when opposite ends of a fragment are inverted tandem repeats of each other, which are especially prevalent when DNA is damaged.

Single-nucleotide polymorphism (SNP) and insertion/deletion (indel) variants detected in each sample were reported in a VCF file, which was submitted to a multilevel filtering. We first used *FilterMutectCalls* function from GATK by setting the minimum AF to 5% (–min-allele-fraction 0.05), the minimum median distance of variants from the end of reads to 3 (–min-median-read-position 3), and enabling “–mitochondria-mode” option. Multiallelic sites were split using bcftools (RRID:SCR_005227, v1.10.2) (Li H. et al., 2009) *norm* function. Subsequently, variants were filtered by testing several parameters in different combinations: the variant allele depth (AD), the total read depth per site (DP), the variant AF, and the contamination rate (CR, previously computed by schmutzi). The patterns of tested filters are shown in Table 1. Consensus sequences were reconstructed using bcftools *consensus* method by inserting only variants with minimum AD = 10 and AF ≥ 0.5 in the mtDNA reference sequence. Finally, we chose to further filter AD10 variants with reference allele depth (RD) = 10 in order to assess potential heteroplasmy.

**TABLE 1:** Patterns of filters tested on the Polizzello dataset.

**CR**	**AD**	**AF**	**DP**
–	–	5	3
–	–	5	–
–	–	50	3
–	–	50	–
–	2	5	4
–	2	5	–
–	2	50	4
–	2	50	–
–	3	5	5
–	3	5	–
–	3	50	5
–	3	50	–
–	5	5	10
–	5	5	–
–	5	50	10
–	5	50	–
–	10	5	20
–	10	5	–
–	10	50	20
–	10	50	–
Mean	–	50	–
Mean	2	50	–
Mean	3	50	–
Mean	5	50	–
Mean	10	50	–
Highest	–	50	–
Highest	2	50	–
Highest	3	50	–
Highest	5	50	–
Highest	10	50	–

*Variants detected by Mutect2 were filtered by testing several parameters in different combinations.CR, contamination rate; AD, variant allele depth; AF, variant allele fraction; DP, total read depth per site.*

### Haplogroup Prediction and Functional Annotation of Variants

Mitochondrial haplogroups were predicted by HaploGrep 2 (Weissensteiner et al., 2016b) *classify* function (v2.2.5) applied to consensus sequences in FASTA format, recalling the Phylotree Build 17 (van Oven and Kayser, 2009b) and applying the default metric (Kulczynski). Haplogroups were assigned to both endogenous sequences generated by schmutzi and consensus sequences obtained by the Mutect2/bcftools pipeline, as described in section “*Materials and Methods*, *Variant Calling and Filtering.”* Graphical phylogenetic trees were also obtained by HaploGrep 2 (web version).

Variant annotations were performed using several resources. Ensembl Variant Effect Predictor (RRID:SCR_007931, web version) (McLaren et al., 2016) provided general annotations about location, impact of variants, and pathogenicity predictions collected by dbNSFP v4.0a (RRID:SCR_005178) (Liu et al., 2016), performed by several software, which included SIFT (RRID:SCR_012813) (Vaser et al., 2016), PolyPhen2 (RRID:SCR_013189) (Adzhubei et al., 2010), DEOGEN2 (Raimondi et al., 2017), FATHMM (Shihab et al., 2013), MutationAssessor (RRID:SCR_005762) (Reva et al., 2011), MutationTaster (RRID:SCR_010777) (Schwarz et al., 2014), PROVEAN (RRID:SCR_002182) (Choi and Chan, 2015), and Condel (RRID:SCR_008584) (González-Pérez and López-Bigas, 2011). Additionally, predictions of the effect of non-synonymous variants by MutPred (RRID:SCR_010778) (Li B. et al., 2009), PANTHER (Thomas and Kejariwal, 2004), PhD-SNP (Altschul et al., 1997), and SNPs&GO (RRID:SCR_005788) (Capriotti et al., 2013) were annotated. Conservation scores for all variant sites by phastCons (Siepel et al., 2005) and phyloP (Pollard et al., 2010) for multiple alignments of 99 vertebrate genomes to the human genome and GERP++ (Davydov et al., 2010) were retrieved from the UCSC Genome Browser,^3^ “Conservation” and “GERP” tracks, respectively. Identifiers for variants were retrieved from dbSNP (Sherry et al., 2001). Other information, including allele frequency in several known datasets and reported associations with diseases, was obtained from databases specialized for mtDNA variants, such as HmtDB (RRID:SCR_007713) (Clima et al., 2017), Mitomap (RRID:SCR_002996) (Lott et al., 2013), and MSeqDR (Shen et al., 2016), and from literature (Diroma et al., 2014, 2016).

### Simulations

We simulated sequencing of aDNA on an Illumina HiSeq 2500 platform using gargammel (Renaud et al., 2017) by randomly choosing the endogenous consensus sequence of one of the Polizzello samples, Pol-7, and its relative contaminant mtDNA consensus sequence, both previously assembled by schmutzi. Single-end reads (option -se) were generated by using the following parameters: endogenous coverage (-c) 100, size distribution with fixed length of fragments (-l) 110, minimum length of fragments (–minsize) 30, read length (-rl) 101. A misincorporation matrix previously computed by mapDamage2.0 on the original sample was provided, specifying double-stranded library (-mapdamage option). We tested different CRs (0.02, 0.1, 0.2, 0.3, 0.4) by changing the argument of the “–comp” option to get FASTQ samples with 2, 10, 20, 30, and 40% present-day human contamination, respectively. Moreover, in order to detect potential heteroplasmies, we generated a simulated sequence of Pol-7 with a coverage = 30X, combined with a simulation of rCRS (coverage = 70X), to obtain a final FASTQ file (coverage = 100X) with different CRs (2, 10, and 20%), where variants should have a hypothetical AF = 30%.

## Results

### Consensus Calling by schmutzi

**FIGURE 2:**
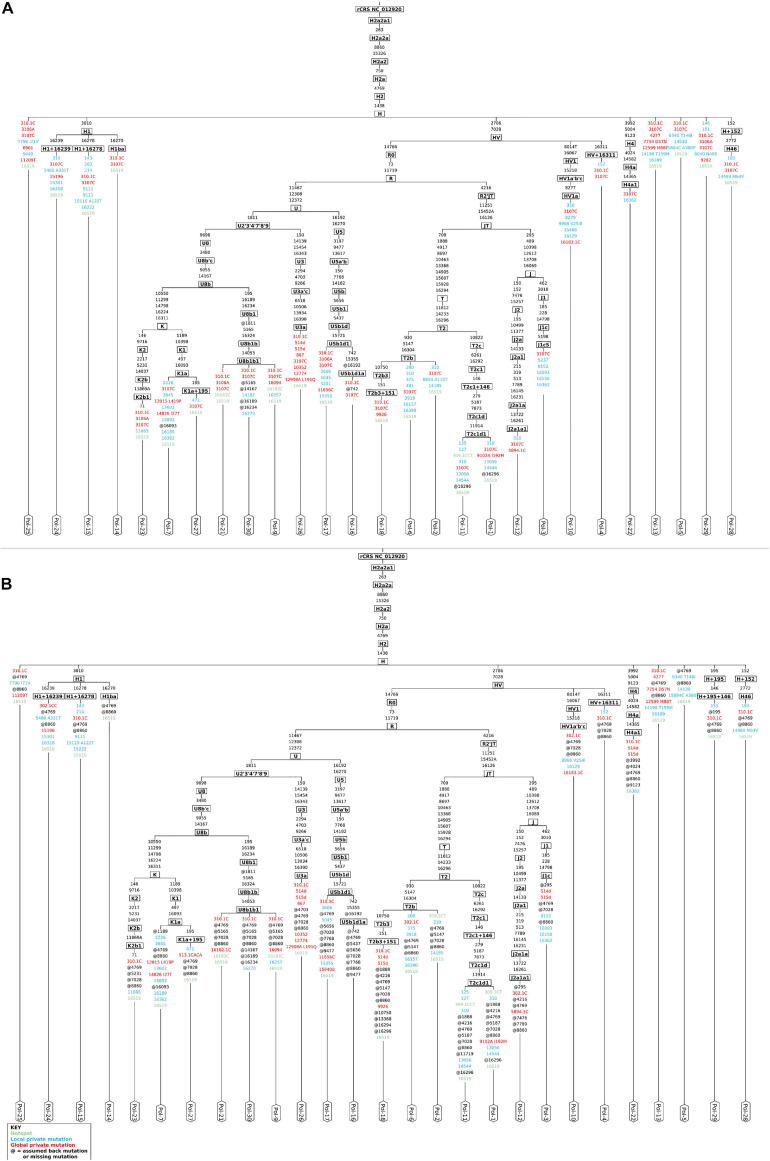
Mitochondrial phylogenetic trees from Polizzello sequences. Phylogenetic trees of mitochondrial DNA variation in Polizzello samples were obtained by Haplogrep2 from consensus sequences generated by schmutzi **(A)** and those obtained from variants called by Mutect2 and retained after filtering based on allele depth (≥10) and allele fraction (≥50%) and considering the highest contamination rate (2%) **(B)**. Slightly different haplogroups were predicted for three samples (Pol-3, Pol-10, Pol-29). Amino acid changes are shown for non-synonymous variants.

The analysis by schmutzi (Renaud et al., 2015) required that all raw reads were aligned onto the sole mitochondrial genome. In this kind of analysis, the realignment to a circular version of the mtDNA by *CircularMapper* is not a mandatory step; however, we chose to perform read realignment to ensure the most accurate haplogroup assignment, since many phylogenetically informative positions can be found at the beginning and the end of the mtDNA reference sequence (Peltzer et al., 2016). schmutzi allowed to identify deamination patterns, which are generally used as markers for aDNA, and then detect recent mitochondrial contaminants by comparing each base position with the corresponding one in 197 known modern Eurasian mtDNA sequences (Renaud et al., 2015). The average CR estimated in each analyzed sample was 0.01, extendible to 0.02 when considering the upper bound of a confidence interval. The good quality of endogenous mitochondrial consensus sequences assembled was confirmed by an accurate mitochondrial haplogroup prediction, involving R0, JT, and U subtrees, all descending from the R clade (Phylotree Build 17), quite in line with their European origin. We were not able to reconstruct any sequence for only three out of 30 samples (Pol-8, Pol-19, Pol-20) due to almost total lack of endogenous mtDNA and too low coverage (mean depth per site = 1.88, 0.24, and 0.19, respectively), thus they were excluded from further analyses. Haplogroups assigned to each sample are shown in Figure 2A. Since the analysis by schmutzi is a well-established practice in the aDNA field, we considered the 27 consensus sequences and the set of related variants detected in each sample as a reference to validate results obtained by our approach.

### Alignment Statistics

**FIGURE 3:**
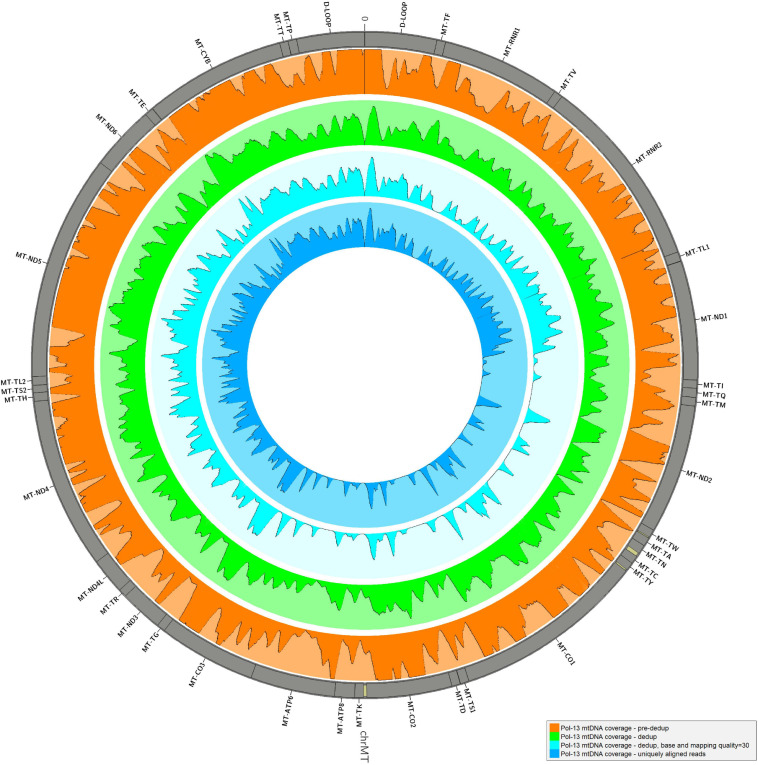
Mean depth of coverage in Pol-13 sample. The mean per base depth of coverage was calculated for Pol-13 alignment at different steps of the pipeline: before and after duplicate removal, filtering by base and mapping quality (≥30), removing all reads ambiguously mapped. Minimum depth in each histogram = 0, maximum depth = 2,000. The graph was generated by Circos (RRID:SCR_011798, v0.69-8) (Krzywinski et al., 2009).

The alignment of reads onto the complete human genome assembly allowed to separate reads mapping to the mtDNA from nuclear DNA ones. Realignment to a circular version of the mtDNA was performed also in this case for the same reason described in *Results*, *Consensus Calling by schmutzi*. After removal of PCR duplicates, which represent artifacts, an average fraction of reads shifting from 1.67%, if single-end, to 3.37% paired-end reads, mapped to both mtDNA and nuclear DNA, thus they were likely belonging to NumtS (Table 2) originating from different portions of the mtDNA genome across evolution. These sequences were even more abundant when considering only deduplicated reads (6.54% in single-end, 16.41% in paired-end reads). Our approach, described in *Methods*, *Variant Calling and Filtering*, allowed to remove possible NumtS sequences and ambiguously mapped reads, thus isolating genuine mtDNA reads with a MQ ≥ 30, whose number per sample is shown in Supplementary Table S1. It is worth noting that, with this protocol, we selected very high confidence reads, since they were all aligned to unique positions with less than 2 mismatches (MQ = 37). The fraction of potential endogenous DNA was calculated for both mtDNA and nuclear DNA in each one of the 30 analyzed samples by dividing the number of uniquely mapped over total sequenced raw reads. On average, we observed a larger enrichment in mtDNA reads generated by paired-end (5.57%) than single-end sequencing (3.05%). On the contrary, more nuclear DNA sequences were obtained by single-end sequencing (12.61% vs. 6.78% by paired-end). As expected, the paired-end sequencing approach allowed a more accurate mapping, as shown not only by the higher fraction of reads uniquely aligned to mtDNA with respect to single-end reads but also by the higher percentage of NumtS detected, probably partially missed by single-end sequencing and mistakenly swapped with pure nuclear sequences. We calculated both nuclear DNA and mtDNA depth of coverage by quantifying read length and read number normalized by genome length: the depth of nuclear DNA, which was captured as off-target sequences, was approximately 0 for almost all samples, while mtDNA sequences were covered from 64.36X on average in single-end samples to 425.73X in paired-end samples (Table 2). Specific regions showed low or no mtDNA coverage, mostly as an effect of PCR duplicates removal and quality filters (Figure 3), and this trend was verified in all samples. Anyway, the mean mtDNA coverage was about 93% when excluding the most fragmented samples (Pol-8, Pol-19, Pol-20). The final mean per base depth of coverage is shown for each sample in Supplementary Figures S1–S6.

**TABLE 2:** Sequencing information of Polizzello samples.

**Sample name**	**Type**	**Endogenous mtDNA (%)**	**Likely NumtS reads (%)**	**Mean per base depth**	**mtDNA coverage (%)**
Pol-1	SE	1.32	0.55	121.18	91.76
Pol-2	PE	5.10	2.98	816.82	97.73
Pol-3	PE	4.00	2.64	369.31	96.47
Pol-4	SE	6.44	3.37	63.93	89.55
Pol-5	PE	6.87	3.93	138.76	96.66
Pol-6	PE	6.69	3.98	511.64	97.79
Pol-7	PE	6.70	3.98	386.28	98.12
Pol-8	PE	0.59	0.47	1.88	62.77
Pol-9	PE	7.16	4.55	319.20	96.67
Pol-10	PE	7.29	4.38	261.09	95.58
Pol-11	SE	0.25	0.13	88.64	90.72
Pol-12	PE	7.17	4.21	284.18	96.30
Pol-13	PE	5.55	3.26	590.39	96.42
Pol-14	PE	5.39	3.24	301.49	95.69
Pol-15	SE	4.52	2.53	65.74	90.48
Pol-16	PE	5.46	3.79	142.82	92.00
Pol-17	SE	5.80	2.87	75.55	91.16
Pol-18	SE	3.30	2.08	64.67	88.74
Pol-19	SE	0.30	0.21	0.24	21.61
Pol-20	SE	0.41	0.34	0.19	15.82
Pol-21	SE	1.47	0.96	56.30	89.57
Pol-22	SE	4.63	2.92	46.83	88.21
Pol-23	SE	3.40	1.91	73.45	90.14
Pol-24	PE	4.94	3.05	315.89	96.42
Pol-25	SE	4.56	2.08	89.86	90.59
Pol-26	PE	5.67	3.09	1, 190.13	98.38
Pol-27	PE	5.86	3.46	804.09	97.93
Pol-28	PE	4.71	2.94	377.70	94.93
Pol-29	SE	5.94	3.24	44.38	88.00
Pol-30	SE	0.33	0.15	110.11	92.58
Average all	**–**	4.39	2.58	257.09	87.63
Median all	**–**	5.02	2.96	129.97	92.29
Average SE	**–**	3.05	1.67	64.36	79.92
Median SE	**–**	3.35	1.99	65.21	89.86
Average PE	**–**	5.57	3.37	425.73	94.37
Median PE	**–**	5.61	3.36	344.26	96.45

*Type of sequencing (PE, paired-end; SE, single-end), mitochondrial DNA (mtDNA) depth, coverage and percentage of potential endogenous mtDNA, and NumtS content are reported for each sample. Endogenous mtDNA percentage was calculated by dividing the number of uniquely mapped reads on mtDNA with mapping quality ≥ 30 with the number of raw reads before Clip&Merge process, while the percentage of likely NumtS reads is the fraction of reads ambiguously mapped to mtDNA and nuclear DNA with respect to the total number of raw reads. The average and median values were calculated for all samples and discriminating single-end from paired-end samples.*

### Outlining a Variant Filtering Pipeline for Mitochondrial DNA Consensus Calling Using Mutect2

Variant calling by GATK Mutect2 (Benjamin et al., 2019) was performed on alignments containing high-quality reads uniquely mapped to mtDNA. A multilevel filtering was then performed considering several factors. The first step consisted of applying the GATK FilterMutectCalls function according to the parameters described in section “*Materials and Methods*, *Variant Calling and Filtering*.” We tested all the patterns of filters (Table 1) related to the number of reads supporting a specific variant allele (AD) and the total number of reads covering a variant position (DP). A further step in the variant filtering process consisted of selecting variant alleles requiring a minimum fraction of total reads per position (AF) to be inserted in a consensus sequence. We chose to set AF ≥ 50%, i.e., include a variant if supported by at least half the total reads covering a specific site in a sample, since it was its major allele and generally confirmed in the corresponding schmutzi consensus sequence. On the other hand, we reported the reference base for sites with AF < 50%, since the variant was the minor allele in those cases. When no AF filter was fixed, 5% was the lowest threshold used to capture information, thus all the detected variants were inserted in the consensus sequence. In case of multiallelic sites, that is, sites with more than one alternative allele in addition to the reference one, in most cases, filters were able to retain only one of the alternatives, if well supported. Otherwise, we preferred to consider reporting the reference allele, since true multiallelic sites are generally not observed very frequently.

**FIGURE 4:**
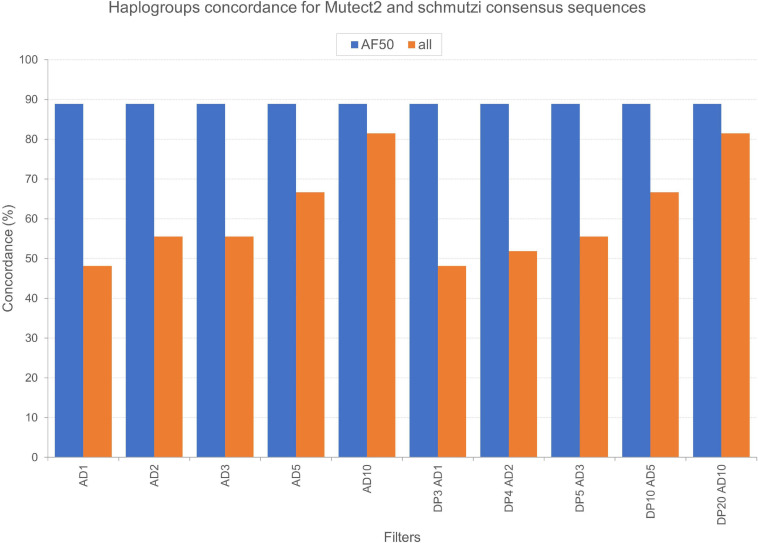
Concordance of haplogroups predicted for Mutect2 and schmutzi consensus sequences of Polizzello samples. The percentage of concordance was calculated on the whole dataset by comparing haplogroups predicted for consensus sequences generated from Mutect2 variants, filtered according to different patterns of filters, to haplogroups assigned to schmutzi consensus sequences. Mutect2 sequences included variant alleles with minimum fraction = 50% (AF50) or alternatively all those detected in non-multiallelic sites (all). AD1, minimum variant allele depth = 1; AD2, minimum variant allele depth = 2; AD3, minimum variant allele depth = 3; AD5, minimum variant allele depth = 5; AD10, minimum variant allele depth = 10; DP3, minimum total depth = 3; DP4, minimum total depth = 4; DP5, minimum total depth = 5; DP10, minimum total depth = 10; DP20, minimum total depth = 20.

We tested the accuracy of our filtering pipeline at two levels: (1) haplogroup-defining variants; (2) all variants in consensus sequences generated from Mutect2 calling. Mitochondrial haplogroups were assigned to all the consensus sequences obtained and compared to the ones predicted for endogenous sequences by schmutzi (Figure 2). In this fashion, we firstly focused on haplogroup-defining variants to evaluate the effect of each one of tested filters: in all cases, the AF threshold ensured the highest percentage of concordance between haplogroups assigned to Mutect2 and schmutzi consensus sequences (88.89%; Figure 4). Even when retaining only variants covered by at least 10 reads (AD = 10), the AF filter allowed to obtain more accurate predictions when enabled (88.89% vs. 81.48%). It is worth noting that each couple of haplogroups differing between schmutzi and Mutect2 consensus sequences of three samples refers to close nodes in the phylogenetic tree (Figure 2). Using more stringent AF filters did not improve the percentage of concordance (AF ≥ 60 or 70 or 80; data not shown). Moreover, the total depth (DP)-based filtering did not reveal any advantage, thus it was excluded from further analyses. Then, we alternately assessed the effect of filtering based on the mean or the highest value of CR, previously computed by schmutzi, with AF ≥ 50% and fixing increasing AD values among those tested. In both cases and for any AD value, no changes were observed in haplogroup predictions, confirming those obtained without specifying a CR value.

Although the CR had no effect on haplogroup-defining variant filtering, we observed a general reduction of the total number of variants when enabling this filtering and varying the CR value (mean or highest; Supplementary Figure S7): on average, from 2.52 (mean rate) to 18 (highest rate) variants were discarded exclusively due to contamination (Supplementary Figure S7B), while 918 and 1,411 variants ca. were filtered out when considering contamination (mean and highest rate, respectively) as one of the reasons (Supplementary Figure S7A). Indeed, Mutect2 internal filtering system also considers other factors affecting the reliability of variants, such as strand bias and weak evidence. In Supplementary Table S2, some statistics generated for each sample are shown, highlighting the prevailing impact of contamination as source of error in variant calling. Also, the AD-based filtering, which did not improve haplogroup concordance as previously reported, had a deep effect on variant filtering, instead, such that could represent a valid alternative to CR parameter in the analysis of our dataset: when the AF ≥ 50% filter was enabled (Supplementary Table S3), the removal of contamination (using both mean and highest CRs) allowed to additionally filter out only variants with a very low coverage (AD = 1) in four samples (Pol-1, Pol-7, Pol-9, Pol-26), while when no AF filter was fixed, the full set of detected variants was slimmed down to a unique subset of reliable variants with or without setting CR, requiring minimum AD = 10 (Supplementary Table S4).

### Quantification of Mitochondrial DNA Variants by Comparing schmutzi and Mutect2 Consensus Sequences

We quantified variants within consensus sequences, discriminating those detected by both methods (schmutzi and Mutect2) and those exclusively detected by one or the other (Supplementary Table S5). Mutect2 detected the same variants reported in schmutzi consensus sequences in large amount (on average, 79.08% of total variants). In the filtering process, we gradually excluded some of these mutational events, leading to a slight decrease in the fraction of variants confirmed by the two methods: 75.91% variants passing Mutect2 filtering step, 75.62% showed AF ≥ 50%, 74.25% covered by at least 10 reads, up to 74.09% variants with both AF ≥ 50% and minimum AD = 10. Overall, about 3.16% of variants reported in schmutzi consensus sequences were discarded since the first step of our filtering pipeline, identified by Mutect2 as artifacts or low-quality variants. Additional filters (AF ≥ 50%, AD ≥ 10) led to even higher percentages, up to 4.98% removed variants. On the other hand, Mutect2 proved to be extremely sensitive, since almost all the variants it called (>98%) were not confirmed by schmutzi consensus sequences. Lots of these variants were then removed due to low-quality base, low AF, strand bias, or contamination or being too close to read ends, reducing to 67.64% variants not confirmed by schmutzi, on average. With further filtering, we ended with a cleaned variant allele set for each sample, of which 3.14% on average did not find evidence in schmutzi sequences. All the percentages for the samples are summarized in Supplementary Table S5.

Since aDNA analysis using schmutzi is a well-established practice, we calculated precision, sensitivity, specificity, and basic statistics related to the variants detected by means of Mutect2 and our filtering protocol, taking as reference variants those inserted by schmutzi in the endogenous consensus sequences (Table 3). We defined as true positives all variants detected by Mutect2 and retained by our filtering process, also detected in schmutzi consensus sequences; true negatives included all non-variant and non-callable sites according to both methods; false positives were reliable variants detected exclusively by Mutect2, while false negatives were variants detected exclusively by schmutzi, without considering those variants in schmutzi sequences discarded by Mutect2 (including a C recurring at position 3107 in all samples, which probably was due to a miscalling around the “N” placeholder at that position of the rCRS reference sequence, thus included in a blacklist of artifacts by Mutect2). Our tested method proved to be a valid alternative, with very high precision (97%), specificity and accuracy (both > 99%), and a low false discovery rate (FDR = 3%), thanks to the usage of strict filters, although at the expense of sensitivity (82% recall rate). It is worth noting that all the false positives in Mutect2 consensus sequences were indel mutations. Moreover, mutated alleles detected by schmutzi at positions 4769 and 8860 were probably intentionally not reported in the VCF generated by Mutect2, being rare polymorphisms in the rCRS sequence, but we did not find any warning about it.

**TABLE 3:** Basic statistics for Mutect2 variants in Polizzello ancient sequences.

**Sample**	**TP**	**TN**	**FP**	**FN**	**PPV**	**SN**	**SPC**	**FNR**	**FPR**	**NPV**	**FDR**	**FOR**	**ACC**
Pol-1	34	16528	1	6	0.97	0.85	1.00	0.15	6.0E-05	1.00	0.03	3.63E-04	1.00
Pol-2	30	16534	1	4	0.97	0.88	1.00	0.12	6.0E-05	1.00	0.03	2.42E-04	1.00
Pol-3	26	16536	2	5	0.93	0.84	1.00	0.16	1.2E-04	1.00	0.07	3.02E-04	1.00
Pol-4	8	16557	0	4	1.00	0.67	1.00	0.33	0.0E+00	1.00	0.00	2.42E-04	1.00
Pol-5	8	16559	0	2	1.00	0.80	1.00	0.20	0.0E+00	1.00	0.00	1.21E-04	1.00
Pol-6	34	16531	1	3	0.97	0.92	1.00	0.08	6.0E-05	1.00	0.03	1.81E-04	1.00
Pol-7	33	16534	0	2	1.00	0.94	1.00	0.06	0.0E+00	1.00	0.00	1.21E-04	1.00
Pol-9	25	16542	0	2	1.00	0.93	1.00	0.07	0.0E+00	1.00	0.00	1.21E-04	1.00
Pol-10	11	16553	1	4	0.92	0.73	1.00	0.27	6.0E-05	1.00	0.08	2.42E-04	1.00
Pol-11	35	16527	0	7	1.00	0.83	1.00	0.17	0.0E+00	1.00	0.00	4.23E-04	1.00
Pol-12	32	16533	1	3	0.97	0.91	1.00	0.09	6.0E-05	1.00	0.03	1.81E-04	1.00
Pol-13	11	16556	0	2	1.00	0.85	1.00	0.15	0.0E+00	1.00	0.00	1.21E-04	1.00
Pol-14	7	16560	0	2	1.00	0.78	1.00	0.22	0.0E+00	1.00	0.00	1.21E-04	1.00
Pol-15	13	16553	0	3	1.00	0.81	1.00	0.19	0.0E+00	1.00	0.00	1.81E-04	1.00
Pol-16	18	16547	0	4	1.00	0.82	1.00	0.18	0.0E+00	1.00	0.00	2.42E-04	1.00
Pol-17	25	16536	1	7	0.96	0.78	1.00	0.22	6.0E-05	1.00	0.04	4.23E-04	1.00
Pol-18	26	16535	2	6	0.93	0.81	1.00	0.19	1.2E-04	1.00	0.07	3.63E-04	1.00
Pol-21	23	16539	1	6	0.96	0.79	1.00	0.21	6.0E-05	1.00	0.04	3.63E-04	1.00
Pol-22	8	16555	3	3	0.73	0.73	1.00	0.27	1.8E-04	1.00	0.27	1.81E-04	1.00
Pol-23	30	16534	0	5	1.00	0.86	1.00	0.14	0.0E+00	1.00	0.00	3.02E-04	1.00
Pol-24	11	16554	1	3	0.92	0.79	1.00	0.21	6.0E-05	1.00	0.08	1.81E-04	1.00
Pol-25	8	16556	0	5	1.00	0.62	1.00	0.38	0.0E+00	1.00	0.00	3.02E-04	1.00
Pol-26	30	16536	0	3	1.00	0.91	1.00	0.09	0.0E+00	1.00	0.00	1.81E-04	1.00
Pol-27	28	16538	1	2	0.97	0.93	1.00	0.07	6.0E-05	1.00	0.03	1.21E-04	1.00
Pol-28	10	16557	0	2	1.00	0.83	1.00	0.17	0.0E+00	1.00	0.00	1.21E-04	1.00
Pol-29	8	16556	0	5	1.00	0.62	1.00	0.38	0.0E+00	1.00	0.00	3.02E-04	1.00
Pol-30	19	16547	0	3	1.00	0.86	1.00	0.14	0.0E+00	1.00	0.00	1.81E-04	1.00
**Mean**	20.41	16544.19	0.59	3.81	0.97	0.82	1.00	0.18	0.00	1.00	0.03	2.31E-04	1.00
**Median**	23.00	16542.00	0.00	3.00	1.00	0.83	1.00	0.17	0.00	1.00	0.00	1.81E-04	1.00

*Basic statistics related to the variants detected in each sample by means of Mutect2 and our filtering protocol [including minimum allele depth (AD) = 10 and minimum allele fraction (AF) = 50%] were obtained by taking as reference variants those inserted by schmutzi in the endogenous consensus sequences. TP, true positives (all variants detected by Mutect2 and retained by our filtering process, also detected in schmutzi consensus sequences); TN, true negatives (all non-variant and non-callable sites according to both methods); FP, false positives (reliable variants detected exclusively by Mutect2); FN, false negatives (variants detected exclusively by schmutzi, without considering those detected by Mutect2 and discarded by our filtering pipeline); PPV, positive predictive value or precision; SN, true positive rate or sensitivity (recall); SPC, true negative rate or specificity; FNR, false negative rate or miss rate; FPR, false positive rate or fallout; NPV, negative predictive value; FDR, false discovery rate; FOR, false omission rate; ACC, accuracy. Mean and median are also reported for the whole dataset.*

### Accuracy of Mutect2 and schmutzi Calls in Simulated Ancient DNA Sequences

The Pol-7 endogenous consensus sequence assembled by schmutzi was randomly chosen as reference for simulations. It contained 36 differences with respect to rCRS, which were reproduced with a hypothetical AF = 100%. We simulated 2, 10, 20, 30, and 40% CRs. New consensus sequences were assembled by schmutzi, all identical to the original sample, and the highest CRs computed were 0.02, 0.02, 0.23, and 0.38, respectively. Mutect2 was then used to call variants in simulated sequences, which were then filtered according to our pipeline, specifying CR computed by schmutzi, and inserted in a consensus sequence when AF ≥ 50%. In all cases, four out of 36 variants were never detected by Mutect2 (m.1189T>C, m.4769A>G, m.7028C>T, and m.8860A>G). One of these variants (m.7028C>T) was instead rightfully detected by Mutect2 in the real Pol-7 sample. Statistics are shown in Table 4. With very low CR (2%), that is, the same computed by schmutzi in Polizzello samples, no artifacts were called by Mutect2. In case of CR = 10%, Mutect2 results were heavily affected by the CR wrongly computed by schmutzi (0.02), leading to FDR = 45%. All the variants miscalled showed low AF (<0.3) and were not included in the consensus sequence. The filtering of Mutect2 calls from data with 10% present-day human contamination was repeated by specifying the correct CR, and only one artifact with AF < 0.2 was detected in this case (FDR = 0.03). The FDR rose to 11 and 64% when contamination reached 20 and 30%, respectively, with all false positives with AF < 0.5, thus artifacts did not affect the consensus sequence. Finally, when CR was set to 40%, four miscalled alleles were inserted in the consensus sequence and the pipeline was not able to filter lots of artifacts (FDR = 63%).

**TABLE 4:** Basic statistics for Mutect2 variants in simulated ancient sequences.

**Simulated CR (%)**	**Tested CR (%)**	**AF (%)**	**TP**	**TN**	**FP**	**FN**	**PPV**	**SN**	**SPC**	**FNR**	**FPR**	**NPV**	**FDR**	**FOR**	**ACC**
2	2	100	32	16533	0	4	1.00	0.89	1.00	0.11	0.0.E+00	1.00	0.00	0.00	1.00
10	2	100	32	16507	26	4	0.55	0.89	1.00	0.11	1.6.E-03	1.00	0.45	0.00	1.00
10	10	100	32	16532	1	4	0.97	0.89	1.00	0.11	6.0.E-05	1.00	0.03	0.00	1.00
20	23	100	32	16529	4	4	0.89	0.89	1.00	0.11	2.4.E-04	1.00	0.11	0.00	1.00
30	27	100	32	16476	57	4	0.36	0.89	1.00	0.11	3.4.E-03	1.00	0.64	0.00	1.00
40	38	100	32	16478	55	4	0.37	0.89	1.00	0.11	3.3.E-03	1.00	0.63	0.00	1.00
2	2	30	31	16533	0	5	1.00	0.86	1.00	0.14	0.0.E+00	1.00	0.00	0.00	1.00
10	2	30	32	16510	23	4	0.58	0.89	1.00	0.11	1.4.E-03	1.00	0.42	0.00	1.00
10	10	30	31	16528	5	5	0.86	0.86	1.00	0.14	3.0.E-04	1.00	0.14	0.00	1.00
20	2	30	31	16458	75	5	0.29	0.86	1.00	0.14	4.5.E-03	1.00	0.71	0.00	1.00
20	20	30	30	16499	34	6	0.47	0.83	1.00	0.17	2.1.E-03	1.00	0.53	0.00	1.00

*Basic statistics related to homoplasmic and heteroplasmic mitochondrial DNA (mtDNA) variants in simulated ancient DNA (aDNA) samples by setting different CRs (2%, 10%, 20%, 30%, and 40%). Variants were detected by Mutect2 and our filtering pipeline by specifying the CR previously computed by schmutzi on simulated samples. For CR wrongly computed by schmutzi, the analysis was repeated with the correct value. CR, contamination rate; AF, allele fraction; TP, true positives (all variants detected by Mutect2 and retained by our filtering process, also detected in schmutzi consensus sequences); TN, true negatives (all non-variant and non-callable sites according to both methods); FP, false positives (reliable variants detected exclusively by Mutect2); FN, false negatives (variants detected exclusively by schmutzi, without considering those detected by Mutect2 and discarded by our filtering pipeline); PPV, positive predictive value or precision; SN, true positive rate or sensitivity (recall); SPC, true negative rate or specificity; FNR, false negative rate or miss rate; FPR, false positive rate or fallout; NPV, negative predictive value; FDR, false discovery rate; FOR, false omission rate; ACC, accuracy.*

We then tried to simulate the same sequence (Pol-7) combined with rCRS in order to generate heteroplasmic variants with an AF = 30% at different levels of contamination (2, 10, and 20%). In a similar way to homoplasmic variant simulation and detection, with very low CR (2%, correctly computed by schmutzi), no artifacts were called by Mutect2 and retained by filtering, with an average AF = 0.29 in line with the expected AF. With increasing levels of contaminant, schmutzi underestimated contamination levels, negatively affecting Mutect2 behavior and determining a high FDR (42 and 71%, respectively). When the correct CR was specified to filter Mutect2 variants, we observed that FDR dropped at 14%, with five artifacts with AF similar to that expected, when CR = 10%, while with a higher level of contamination (20%), the possibility to detect true heteroplasmic variants was under 50%.

### Annotation of Filtered Mutect2 Mitochondrial DNA Variants and Assessment of Potential Heteroplasmy

**FIGURE 5:**
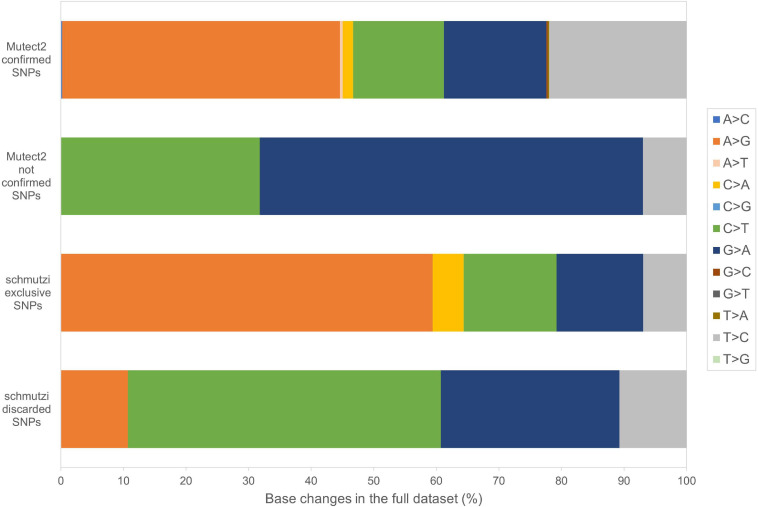
Base changes composition in Mutect2 and schmutzi variants. All the possible base changes were quantified for the whole dataset considering variants detected by Mutect2, reliable according to our filtering pipeline, confirmed by both methods (Mutect2 and schmutzi), those exclusively detected by one or the other, and schmutzi variants we discarded (detected by Mutect2 but not passing filtering).

**FIGURE 6:**
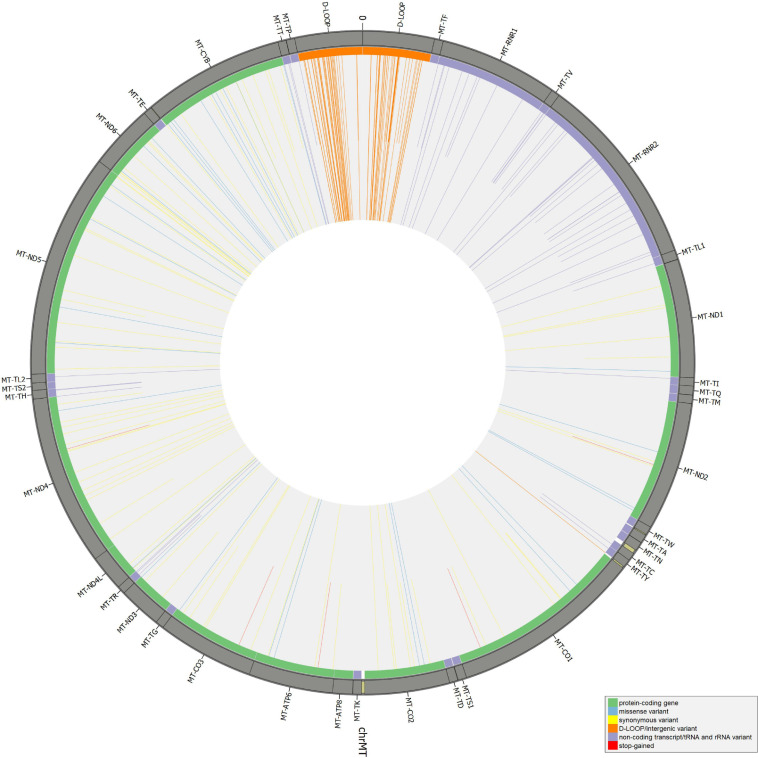
Distribution of mitochondrial DNA (mtDNA) variants in the whole Polizzello dataset. Longer bars refer to variants above the allele fraction (AF) threshold (50); shorter bars refer to variants under the threshold (possible low-to-mid heteroplasmies).

Beyond mutational events that were inserted in consensus sequences, other variants detected by Mutect2 were retrieved since passing all the filters previously described, leaving aside AF, useful only for assembling consensus sequences. In Supplementary Table S6, the total number of variants was reported for each sample, separating SNPs and indels and specifying all the possible base substitutions. Multiple nucleotide polymorphisms were rarely detected. The transition to transversion ratio was calculated for 16 out of 27 samples due to a complete lack of transversions in the remaining 11 samples. On average, this ratio was higher than the expected value for human mtDNA (25.25 vs. 15) (Tamura and Nei, 1993), highlighting a strong transition bias, which was also observed in 19 of the 27 schmutzi consensus sequences. As expected in aDNA (Binladen et al., 2006), type 1 (A > G, T > C) and type 2 (C > T, G > A) transitions were the most frequent events observed due to nucleotide misincorporation. Although we could not extend the classification of variants identified by our protocol (true and false positives, true and false negatives) to those with AF < 50%, since they were not expected to be in a consensus sequence, being minor alleles, we surveyed the composition of base changes for all variants detected by Mutect2, considered reliable according to our filtering pipeline and confirmed by both methods (Mutect2 and schmutzi), those exclusively detected by one or the other, and schmutzi variants we discarded (Figure 5). Both methods were able to detect disparate mutational events, although A > G and T > C covered more than half of them (66.39%). A prevalence of G > A, C > T, and T > C events was observed for variants exclusively detected by Mutect2, while A > G changes were the most missing type with respect to schmutzi variants. The 50% of variants in schmutzi sequences discarded by our filtering pipeline were C > T substitutions. All the variants detected by fixing minimum AD = 10 and CR = 0.02 were annotated using several resources (Supplementary Table S7), including specific mtDNA databases, such as Mitomap (Lott et al., 2013), HmtDB (Clima et al., 2017), and MSeqDR (Shen et al., 2016), which are a benchmark for modern mtDNA analysis. The whole set included 282 DNA variants, 10 of which were insertions, three deletions, and 269 SNPs, whose distribution is shown in Figure 6. Variants clustered quite uniformly among intergenic (28% ca., mainly within the D-Loop), missense (26%), and synonymous (26%) variants, followed by non-coding transcript variants (rRNAs and tRNAs, 18%, Supplementary Figure S8A, inner circle). A small fraction (2%) of stop gained variants was also detected as possible low heteroplasmies, thus the onset of a disease due to these variants was very unlikely. Variants with AF ≥ 50% included six insertions, two deletions, and 175 SNPs and prevailed in intergenic regions (almost 38%, Supplementary Figure S8A, outer circle), followed by protein-coding genes (synonymous variants 33% ca., missense variants 18%), while those in non-coding transcript genes were detected to a lower extent (12%). Variant frequency normalized to the length of each mtDNA locus highlighted the greatest spread in the D-Loop, followed by some tRNA coding genes, while among protein-coding genes, MT-ND6 and MT-CYB were the most variable considering both the whole set of variants and the subset of variants inserted in consensus sequences (Supplementary Figure S8B). The most detected variants (182) were singletons, i.e., variants detected only in one sample, while four variants with an AF ranging between 84 and 99% were shared by all samples and confirmed as common polymorphisms in several reference datasets [mean frequency > 96% in Mitomap, >97% in HelixMTdb (Bolze et al., 2020), >91% in HmtDB]. Interestingly, 19 out of 282 variant alleles, mostly recurrent in our dataset, corresponded to ancestral alleles from the Reconstructed Sapiens Reference Sequence (RSRS), an inferred sequence from both a global sampling of modern human samples and samples from ancient hominids (Behar et al., 2012). Globally, among variants shared by at least two samples, six out of 19 missense variants, which generally have a moderate impact on cell functionality, were possible high-heteroplasmies or quasi-homoplasmies associated with diseases and/or predicted as pathogenic by more than one software (see section “*Materials and Methods”*) (*MT-ND1* m.4216T>C in Pol-2, Pol-3, and Pol-6; *MT-ND2* m.4917A>G in Pol-1, Pol-2, Pol-6, Pol-11, and Pol-18; *MT-CO1* m.6261G>A in Pol-1 and Pol-11; *MT-ATP6* m.9055G>A in Pol-7, Pol-9, Pol-21, Pol-23, Pol-27, and as medium heteroplasmy in Pol-30; *MT-ND3* m.10398A>G in Pol-3, Pol-7, Pol-12, and Pol-27; *MT-ND5* m.13708G>A in Pol-3 and Pol-12); the remaining were potential low heteroplasmies or variants predicted as neutral.

**FIGURE 7:**
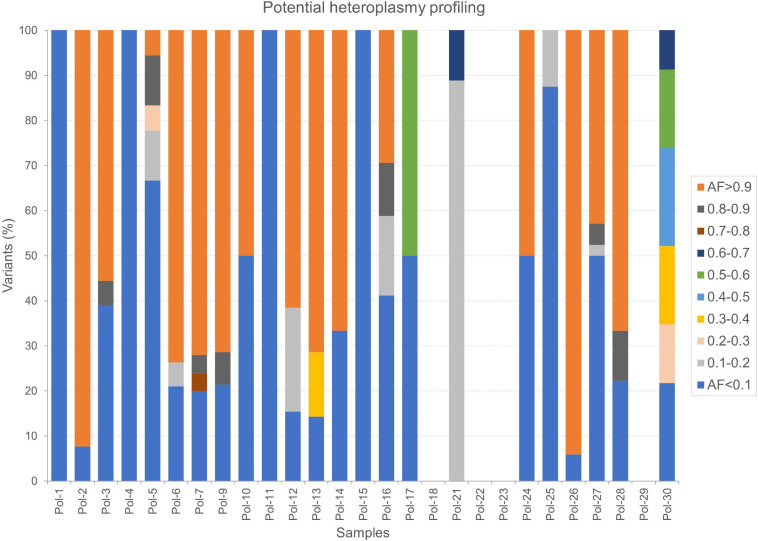
Potential heteroplasmy profiles in Polizzello samples. The percentage of variants in each sample was calculated considering all the ranges of variant allele fraction. Here, 5% was the lowest threshold used to capture information.

Finally, we thoroughly surveyed AFs by considering only variant sites where the reference allele was also detected and supported by as much high number of reads by fixing both minimum variant AD and RD equal to 10. In this fashion, we selected potential heteroplasmies, classified according to different ranges of AF and base changes (Supplementary Table S8). A deep transition bias was further stressed in this subset of variants. The most abundant classes were potential low heteroplasmies (AF < 10%, on average 3.59 per sample) and quasi-homoplasmies (AF ≥ 90%, on average 4.96 per sample), while few variants covered all other ranges. A profile of heteroplasmy was obtained for our dataset (Figure 7), highlighting other prevailing AFs in each sample, particularly 10–20% and 50–60%. Since stringent filters were set in this analysis, the possibility to detect heteroplasmy seemed strongly correlated to mtDNA depth of coverage (Table 2). Indeed, no variants passed the required filters in four samples (Pol-18, Pol-22, Pol-23, and Pol-29), whose mean mtDNA depth of coverage was < 100X, which could be considered as the lower limit to accurately detect potential heteroplasmies. However, we cannot assume this as a general trend, since, being an average value, some parts of the genome can be better covered than the remaining ones. Indeed, a small number of high confident partially mutated sites were retrieved even with lower mean per base depth values (e.g., 56.30 in Pol-21), as well as a quite variegated overview in terms of heteroplasmy composition was observed in other samples with coverage not so much higher than 100X (e.g., 110.11 in Pol-30; Figure 7).

## Discussion

In this work, we implemented a pipeline for ancient mtDNA analysis, which exploits specific peculiarities of several tools, namely, mapDamage2.0 (Jónsson et al., 2013), schmutzi (Renaud et al., 2015), and GATK Mutect2 (Benjamin et al., 2019). While the first two software are commonly used in the field of aDNA studies in order to estimate deamination patterns and CRs, respectively, Mutect2 is a quite recent software, initially developed with the aim to detect somatic mutations in tumor samples and then adapted to mtDNA analysis. Indeed, an *ad hoc* variant caller is required to conceive not only a condition differing from both the haploid and diploid ones, due to the presence of several copies of mtDNA, but also the possibility of the coexistence of different mtDNA genotypes within the same cell, tissue, or individual, a condition known as heteroplasmy. Identifying heteroplasmic mtDNA variants and establishing heteroplasmy levels in ancient samples are not trivial tasks due to the damaged nature of most aDNA samples and contamination with exogenous DNA either from other species or from modern humans. To the best of our knowledge, no previous study used Mutect2 for ancient mtDNA analysis as well as no survey was thoroughly performed on AFs of mtDNA variants in ancient samples. In this work, we evaluated the efficacy of Mutect2 for variant calling in aDNA by introducing its usage in a pipeline we developed, outlined in Figure 1, available as Supplementary Data. To this aim, firstly, nearly complete (≥ 88%) mtDNA consensus sequences were assembled for most samples (27 out of 30) after the detection and filtering of mtDNA variants with an AF high enough to expect a functional effect, although the mutation load threshold above which a mutation can exert a phenotypic effect may vary depending on the type of change and tissue (Rossignol et al., 2003; Gasparre et al., 2011; Chinnery and Hudson, 2013). We fixed a minimum threshold of AF to 50%, allowing to insert the prevailing allele for each genomic position in the consensus sequence obtained for each sample. All the variants above this threshold were compared with those detected by a well-used tool in the aDNA field, schmutzi. However, because of the complexity of mtDNA due to its ploidy and heteroplasmy, we cannot disregard mtDNA variants under the fixed threshold, even if identifying them as true variants, sequencing artifacts or contaminations is not straightforward. To this aim, a thorough annotation of all the variants after a stringent filtering was performed, gathering as much information as possible from well-known and specialized databases (Sherry et al., 2001; Lott et al., 2013; Shen et al., 2016; Clima et al., 2017).

The pipeline we developed included some steps, such as adapter removal, realignment for circular genomes, and removal of PCR duplicates, which are generally highly recommended for NGS data as well as a common practice in other pipelines for aDNA analysis, such as EAGER (Peltzer et al., 2016). For each sample, we quantified the fraction of pure mtDNA reads, nuclear DNA reads, unmapped reads, PCR duplicates, and reads mapped ambiguously to mtDNA and nuclear DNA. These latter can be linked to potential contamination of NumtS, nuclear fragments derived from mtDNA during evolution (Lopez et al., 1994; Hazkani-Covo et al., 2010; Simone et al., 2011; Calabrese et al., 2017; Bücking et al., 2019), which can confound the phylogenetic reconstructions (van der Kuyl et al., 1995; Willerslev and Cooper, 2005) and affect the detection of true variants (Yao et al., 2008; Maude et al., 2019) in modern as well as ancient mtDNA samples. These nuclear sequences are retrieved in mtDNA-targeted sequencing due to their high similarity with mtDNA and to a certain aspecificity of probes, thus their removal is crucial in any case, whether they are true endogenous NumtS or NumtS belonging to a contaminant sample. As the stoichiometric ratio between the mitochondrial and nuclear genome within cells generally favors the mitochondrial one, the NumtS issue has generally been neglected in aDNA studies as well as by specialized pipelines, such as schmutzi, whose approach avoids large gaps of coverage in mtDNA alignments. Despite mitochondria isolation and enrichment, NumtS amplification in aDNA sequencing can still be possible (den Tex et al., 2010), thus computational methods for NumtS identification also in aDNA samples have been developed (Samaniego Castruita et al., 2015). In order to exclude putative NumtS, a regular method based on using only reads mapping uniquely to a genomic reference that contains the nuclear and the mitochondrial genomes together resulted feasible for humans (Picardi and Pesole, 2012; Calabrese et al., 2014; Santibanez-Koref et al., 2019) and became a recommended step also in aDNA studies (Peltzer et al., 2016). We embraced this methodology and selected uniquely mtDNA mapped reads through base and MQ filters generally used in aDNA studies (Posth et al., 2016; Modi et al., 2017). In this fashion, we preferred to retain high-quality sequences at the risk of loss of coverage, always observed in the same specific regions in all our samples, and consequent generation of fragmented sequences. Another essential step in our pipeline was base quality score downscaling of likely damaged positions in the reads by mapDamage2.0, which allows to quantify DNA damage patterns in ancient sequences. After variant calling by Mutect2, several filters were tested to identify the most useful and proper parameters for our dataset. A variant filtering based on the total depth of each covered genomic position proved to be ineffective in most cases or too much stringent. For consensus sequence assembly, we preferred to select variant alleles absolutely and relatively well supported by fixing minimum variant AD and AF, respectively. The value chosen as minimum AD (10) is generally too high for aDNA sequences; however, it was convenient in our case, thanks to a high sequencing coverage. Moreover, contamination was estimated to such a low rate in our dataset (maximum 2% computed by schmutzi) that adding this information when using GATK *FilterMutectCalls* function had no effect on our consensus sequences. We proved the relevance of setting a contamination estimate when filtering Mutect2 variants under the fixed threshold of AF and generally when higher CRs were simulated (40%). The comparison of consensus sequences generated by our pipeline with those assembled by schmutzi highlighted great accuracy in the detection of both haplogroup- and non-haplogroup-defining variants, which were almost totally confirmed (on average 20 in each sample). Indeed, despite assembled sequences were not complete, predicted haplogroups were mostly concordant with those assigned to schmutzi consensus sequences, and the few haplogroups discording were very close nodes in the phylogenetic tree (van Oven and Kayser, 2009b). Rare Mutect2 calls passing filters were not reported in schmutzi consensus sequences, while on the other hand, we were able to quantify a certain number of variant alleles exclusively detected by schmutzi (on average six per sample). These latter can be minimally attributed to differences in the algorithm for variant calling, while most of them were, actually, also detected by Mutect2, but then discarded according to our filtering due to low depth or low quality. We observed poor advantage in variant filtering by setting lower AD values (1, 2, 3, or 5) with consequent gain of sensitivity to the detriment of specificity, since a few SNPs were additionally retrieved with respect to those detected by schmutzi, together with an increase of variants exclusively detected by Mutect2. Moreover, about the 50% of variants in schmutzi sequences discarded by our filtering pipeline were C > T substitutions, typical markers of aDNA, probably due to the quality score rescaling effect by mapDamage2.0, where a different algorithm than the one implemented in schmutzi is used to quantify deamination.

The comparison of our results with those obtained using schmutzi was possible only for Polizzello consensus sequences and reproduced with simulated aDNA sequencing data with coverage 100X, which highlighted great accuracy in the generation of consensus sequences even when the contamination reached higher levels, up to 30%. To assess the accuracy of Mutect2 variants with lower AF than those inserted in consensus sequences, additional simulations were performed, reproducing heteroplasmies at different CRs (2, 10, and 20%). We fixed AF = 30%, since it could represent a low-to-mid heteroplasmy level. Our pipeline proved to be quite efficient in discriminating true endogenous variants from artifacts or contaminations when two conditions were verified: (1) CR was correctly computed by specific tools (schmutzi in our case) and (2) low levels of contamination were detected (FDR = 0 when CR = 2%, FDR = 14% when CR = 10%). Since the Polizzello samples we were able to assemble seemed to show only 2% of contamination according to schmutzi, all the filtered variants detected by Mutect2 could be highly reliable and reveal true heteroplasmy. We further filtered variant positions where both variant and reference alleles were highly covered (at least 10 reads for both), leading to an enrichment in quasi-homoplasmies and very low heteroplasmies, with a strong transition bias. We have to say that besides the contamination issue, which affects miscalling rates, the other main feature concerning aDNA samples is postmortem damage, with an increase of specific types of misincorporations, that are A > G, T > C (both type 1), C > T, G > A (both type 2) transitions due to deamination and G > T transversions linked to depurination. These latter were never observed in our dataset, while the reliability of transitions depends greatly on their positions in the sequencing reads (Briggs et al., 2007). A drastic resolution consisted of removing all transitions (Axelsson et al., 2008) but proved to be inappropriate (Rambaut et al., 2009) as well as unfeasible in our case, since only transitions were detected in many of our samples (11 out of 27). To this aim, we considered 12 bases from each side by both schmutzi and mapDamage2.0, thus deaminations were properly evaluated in the variant calling process. Nevertheless, the transition to transversion ratio was far from the expected values in both Mutect2 and schmutzi results, partially explicable by gaps of coverage and possibly by deamination events unforeseen by existing algorithms for misincorporation pattern estimate.

Disease annotations, conservation scores, pathogenicity predictions, together with allele frequencies in known databases, which generally provide a useful tool to discriminate between known and novel variants and between neutral and deleterious variant alleles in modern mtDNA, can be used in aDNA analysis to evaluate variants (Toncheva et al., 2020), e.g., making it possible to discover variants lost in modern humans due to purifying selection. Significantly, about 7% of variants mostly recurrent in our dataset corresponded to ancestral alleles from the RSRS (Behar et al., 2012), and more than the half of them (10 out of 17 sites) were also highly frequent in modern mtDNA databases, including four of the seven rare polymorphisms in rCRS, suggesting that a variant calling without reference allele bias was performed.

## Conclusion

We proved the high reliability of mtDNA variants from ancient samples using expedients to avoid any kind of contaminations, thanks to a customized pipeline including variant calling, filtering, and annotation. With sufficient coverage levels, the usage of GATK Mutect2 for variant calling in mitochondrial aDNA allows to exploit peculiar features of NGS data in order to look beyond consensus sequences and examine also true partially mutated sites, when CRs are correctly estimated by external tools. Additional analyses including larger datasets of ancient mtDNA sequences are required to provide novel insights on aDNA and the mitochondrial disease spectrum in ancient times. Further information about age and kinships can be exploited to detect private variants in families and infer hereditary diseases. The availability of public data reporting all variants detected in ancient mtDNA sequences, together with sequencing information as reported, e.g., in the VCF format offered by Mutect2, could provide novel stimulus in the challenging analysis of mitochondrial heteroplasmy in ancient humans.

## Data Availability Statement

The dataset generated for this study can be found in the NCBI Sequence Read Archive (SRA) database under Accession Number PRJNA669657. Consensus sequences in FASTA format are available in GenBank under Accession Numbers MW389247–MW389273. All other relevant data are available upon request.

## Ethics Statement

Ethical review and approval was not required for the study on human participants in accordance with the local legislation and institutional requirements. Written informed consent for participation was not required for this study in accordance with the national legislation and the institutional requirements.

## Author Contributions

MAD, AM, and SV conceived the research. MAD performed the bioinformatics analyses, pipeline implementation, and data visualization and drafted the manuscript. AM performed the laboratory work. LS analyzed and selected the archeological material. DC and ML provided the reagents and materials. MAD, AM, and SV reviewed the manuscript with input from all co-authors. All the authors read and approved the final manuscript.

## Conflict of Interest

The authors declare that the research was conducted in the absence of any commercial or financial relationships that could be construed as a potential conflict of interest.

## Footnotes

1 FastQC, a Quality Control tool for High Throughput Sequence Data. Babraham Bioinformatics http://www.bioinformatics.babraham.ac.uk/projects/fastqc/

2 Picard Tools – By Broad Institute. https://broadinstitute.github.io/picard/

3 UCSC Genome Browser – Table Browser. https://genome.ucsc.edu/

## Abbreviations

aDNA: ancient DNA
mtDNA: mitochondrial DNA
C to T (or C > T): cytosine to thymine
G to A (or G > A): guanine to adenine
NumtS: nuclear mitochondrial sequence
NGS: next-generation sequencing
VCF: Variant Call Format
rCRS: revised Cambridge Reference Sequence
BAM: Binary Sequence Alignment/Map
SNP: single-nucleotide polymorphism
indel: insertion/deletion
AD: variant allele depth
DP: total read depth
AF: allele fraction
CR: contamination rate
RD: reference allele depth
MQ: mapping quality
FDR: false discovery rate
A > G: adenine to guanine
T > C: thymine to cytosine
rRNA: ribosomal RNA
tRNA: transfer RNA
RSRS: Reconstructed Sapiens Reference Sequence.
