# Supplementary material for: New Insights Into Mitochondrial DNA Reconstruction and Variant Detection in Ancient Samples
Source: Front Genet. 2021 Feb 18;12:619950. doi: 10.3389/fgene.2021.619950 (PMC7930628; doi:10.3389/fgene.2021.619950)
Supplement: Supplementary file 1 [file Data_Sheet_1.PDF]

Supplementary Material

1 Supplementary Data

Command lines used for the analysis

adapter removal and read merging by Clip&Merge (EAGER)

#single-end reads

```
ClipAndMerge -in1 input_file.fastq.gz -f
AGATCGGAAGAGCACACGTCTGAACTCCAGTCAC -r
AGATCGGAAGAGCGTCGTGTAGGGAAAGAGTGTA -trim3p 0 -trim5p 0 -l 30 -qt -
q 30 -log clip_SE.log -m 1 -o clipped.fastq.gz
```

#paired-end reads

```
ClipAndMerge -in1 input_file_R1.fastq.gz -in2
input_file_R2.fastq.gz -f AGATCGGAAGAGCACACGTCTGAACTCCAGTCAC -r
AGATCGGAAGAGCGTCGTGTAGGGAAAGAGTGTA -trim3p 0 -trim5p 0 -l 30 -m 1
-qt -q 30 -p 10 -log merge.log -o clipped_merged.fastq.gz -u
forwards.unmerged.fq.gz reverse.unmerged.fq.gz
```

read alignment onto hg19 reference genome assembly (replace GRCh37.fasta with rCRS.fasta for schmutzi analysis)

mtDNA elongation

```
circulargenerator -e 500 -i GRCh37.fasta -s MT
```

read alignment by BWA (same commands for clipped.fastq.gz)

```
bwa aln -t 4 GRCh37_500.fasta clipped_merged.fastq.gz -n 0.01 -l
16500 -f out.sai 2> aln.err
```

```
bwa samse -r "@RG\tID:sample\tSM:sample\tPL:illumina" -f aln.sam
GRCh37_500.fasta aln.sai clipped_merged.fastq.gz 2> aln.samse.err
```

realignment to mtDNA by CircularMapper (EAGER)

```
realignsamfile -e 500 -i aln.sam -r GRCh37.fasta 2>
realignment.err
```

aligned read processing and filtering

part 1 (also followed for schmutzi analysis)

```
samtools view -@ 4 -b -o mapped.bam realigned.bam
```

```
samtools sort -@ 4 -m 8G -o mapped.sorted.bam mapped.bam

samtools index mapped.sorted.bam

picard CleanSam INPUT=mapped.sorted.bam OUTPUT=cleaned.bam
VALIDATION_STRINGENCY=SILENT 2> cleaning.err

makedir sample_dedup

dedup -i cleaned.bam -o sample_dedup 2> dedup.err

cd sample_dedup

samtools sort -@ 4 -m 8G -o cleaned_rmdup.bam.sorted.bam
cleaned_rmdup.bam

samtools index cleaned_rmdup.bam.sorted.bam
```

part 2

```
samtools view -h -q 30 -F 4 -F 256 -F 2048
cleaned_rmdup.bam.sorted.bam "MT:1-16569" | grep -v -P
'XA:Z:[^\tMT]+' |  grep -v SA:Z | awk 'BEGIN {FS="\t"}
{if($0~/X1:i:0/||$0~/^@/)print $0}' | samtools view -b - >
mt_q30.bam

samtools view cleaned_rmdup.bam.sorted.bam "MT:1-16569" | grep -P
'XA:Z:[^\tMT]+' > potential_NumtS.sam

samtools view cleaned_rmdup.bam.sorted.bam | awk 'BEGIN {FS="\t"}
{if ($3!="MT") {print $0}}' | grep -P 'XA:Z:[\tMT]+' >>
potential_NumtS.sam

samtools view -q 30 -F 4 -F 256 -F 2048
cleaned_rmdup.bam.sorted.bam | awk 'BEGIN {FS="\t"} {if ($3!="MT")
{print $0}}'| grep -v -P 'XA:Z:[^\tMT]+' |  grep -v SA:Z | awk
'BEGIN {FS="\t"} {if($0~/X1:i:0/||$0~/^@/)print $0}' > nu_q30.bam
```

schmutzi analysis (can be replaced with other tool specific to calculate contamination rate; BAM file obtained from aligning sample to rCRS.fasta and then processed as above)

```
samtools calmd -b mt_cleaned_rmdup.bam.sorted.bam rCRS.fasta >
mt_MD.bam

samtools index mt_MD.bam

contDeam.pl --lengthDeam 12 --library double --out mt_MD_contDeam
--uselength --ref rCRS.fasta mt_MD.bam 2> contDeam.err

schmutzi.pl --lengthDeam 12 --out mt_MD_schmutzi_out --ref
rCRS.fasta --t 20 mt_MD_contDeam
/path/to/schmutzi/alleleFreqMT/197/freqs/ mt_MD.bam 2>
schmutzi.err
```

deamination estimate, quality score rescaling by mapDamage2.0

```
mapDamage -i mt_q30.bam -r GRCh37.fasta -l 101 -d
sample_mapDamage_output --rescale --rescale-out=rescaled.bam 2>
mapdamage.err
```

```
samtools index rescaled.bam
```

```
samtools index rescaled.bam
```

variant calling by GATK Mutect2

```
gatk Mutect2 --input rescaled.bam --output mt.vcf --reference
GRCh37.fasta -L MT --mitochondria-mode --initial-tumor-lod 0 --
tumor-lod-to-emit 0 --af-of-alleles-not-in-resource 4e-3 --
pruning-lod-threshold -4 --annotation UniqueAltReadCount --
showHidden 2> mutect2.err
```

variant filtering

variant filtering by GATK (specify the contamination rate previously calculated by schmutzi (or other tool) as argument of the parameter .--contamination-estimate)

```
gatk FilterMutectCalls --variant mt.vcf --output mt_filtered.vcf -
-reference GRCh37.fasta -L MT --max-alt-allele-count 1 --min-
allele-fraction 0.05 --min-median-read-position 3 --mitochondria-
mode --autosomal-coverage 0.01 --filtering-stats
mt_filtering.stats --stats mt.stats --contamination-estimate 0.01
--showHidden 2> gatk_filtering.err
```

vcf processing and further variant filtering

```
cat <(grep ^# mt_filtered.vcf) <(grep -v ^# mt_filtered.vcf | grep
PASS) >> mt_filtered_PASS.vcf
```

```
bcftools norm -m -any -O vcf -o mt_filtered_PASS_split_multi.vcf
mt_filtered_PASS.vcf 2> split.err
```

```
cat <(grep ^# mt_filtered_PASS_split_multi.vcf) <(grep -v ^#
mt_filtered_PASS_split_multi.vcf | awk -F '\t' '$10~/([0-
9]([/|])+[0-9])+:[0-9]*,([1-9]+[0-9]+)+:.+/') >>
mt_filtered_PASS_split_multi_AD10.vcf
```

```
cat <(grep ^# mt_filtered_PASS_split_multi_AD10.vcf) <(grep -v ^#
mt_filtered_PASS_split_multi_AD10.vcf | awk -F '\t' '$10~/([0-
9]([/|])+[0-9])+:([1-9]+[0-9]+)+,([1-9]+[0-9]+)+:.+/') >>
mt_filtered_PASS_split_multi_AD10_RD10.vcf
```

```
cat <(grep ^# mt_filtered_PASS_split_multi.vcf_AD10.vcf) <(grep -v
^# mt_filtered_PASS_split_multi_AD10.vcf | awk -F '\t' '$10~/([0-
9]([/|])+[0-9])+:[0-9]*,[0-9]*:0\.[5-9][0-9]*:.+/') >>
mt_filtered_PASS_split_multi_AD10_AF50.vcf
```

```
bgzip mt_filtered_PASS_split_multi_AD10_AF50.vcf

tabix -p vcf mt_filtered_PASS_split_multi_AD10_AF50.vcf
```

consensus sequence assembly and haplogroup prediction

```
samtools faidx GRCh37.fasta MT:1-16569 | bcftools consensus
mt_filtered_PASS_split_multi_AD10_AF50.vcf.gz > mt_AD10_AF50.fasta
2> consensus.err

sed -i "s/MT:1-16569/sample_name/g" mt_AD10_AF50.fasta

haplogrep classify --extend-report --format fasta --in
mt_AD10_AF50.fasta --out haplogroup_mt_AF50_AD10.txt --phylotree
17 2> haplogrep.err
```

2 Supplementary Figures

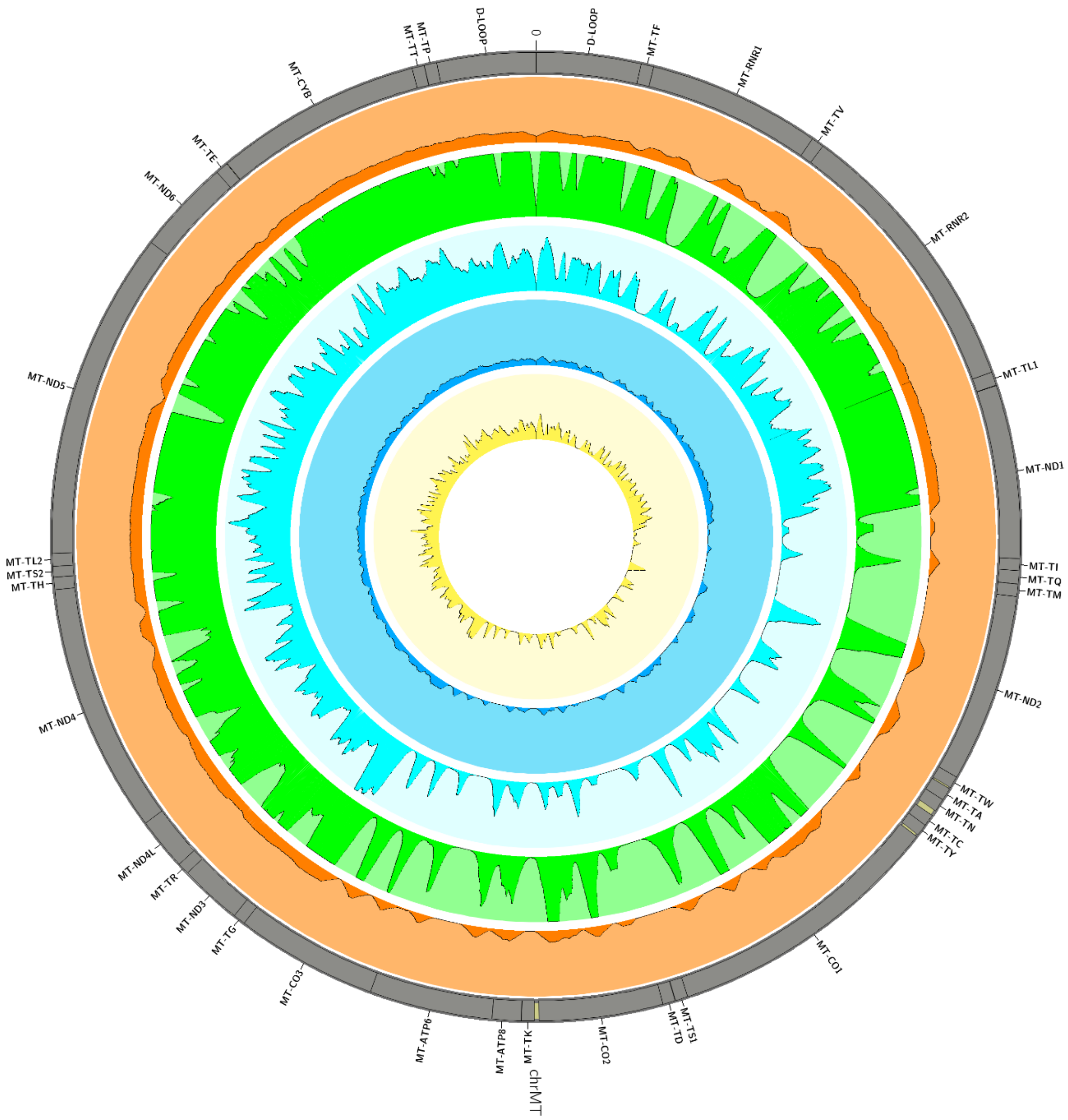

Figure S1. Mean mtDNA depth of coverage. The mean per base depth of coverage was calculated for Polizzello samples. Minimum depth in each histogram = 0, maximum depth = 1000. Color legend for histograms: orange = Pol-1; green = Pol-2; cyan = Pol-3; blue = Pol-4; yellow = Pol-5.

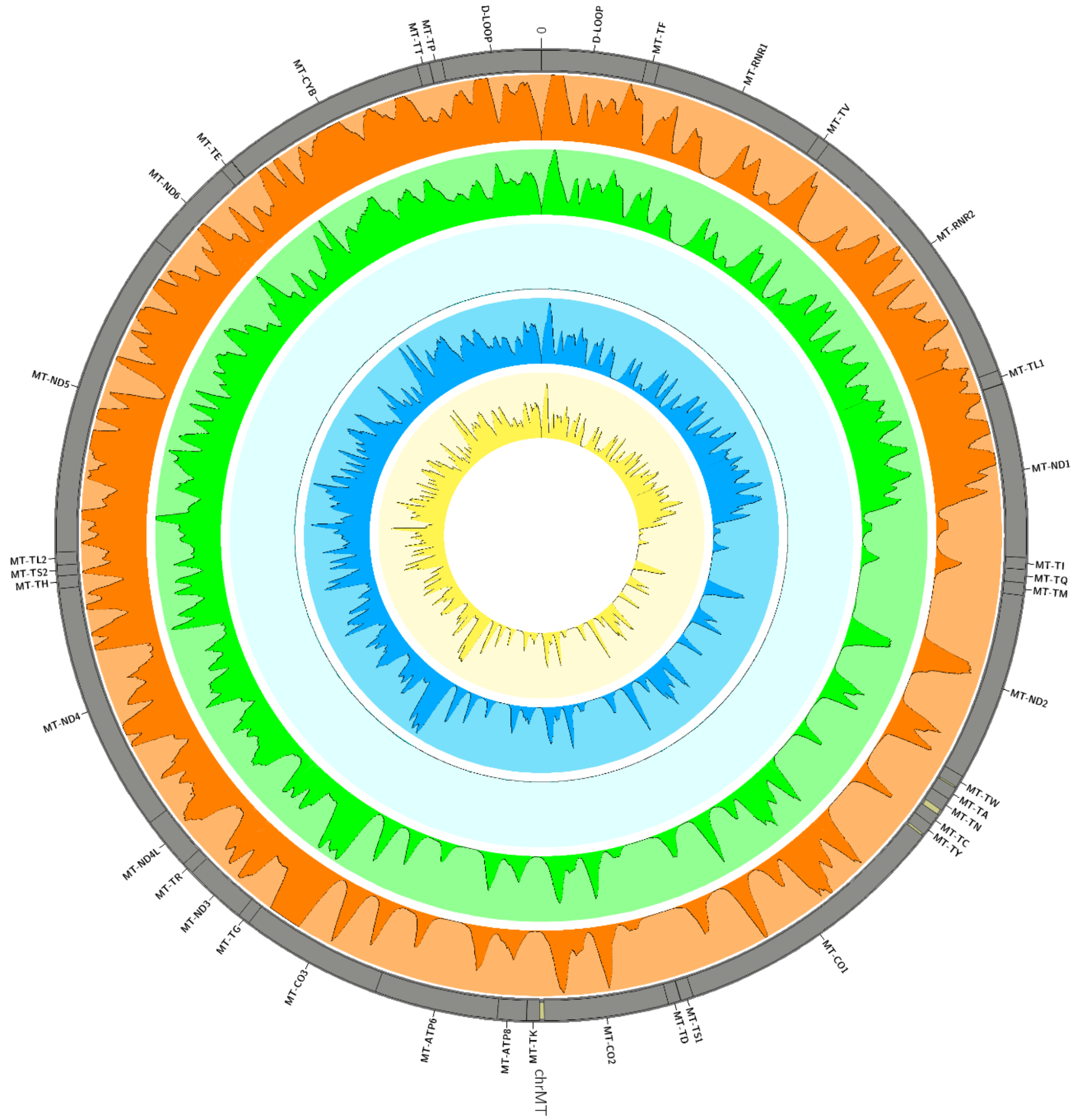

Figure S2. Mean mtDNA depth of coverage. The mean per base depth of coverage was calculated for Polizzello samples. Minimum depth in each histogram = 0, maximum depth = 1000. Color legend for histograms: orange = Pol-6; green = Pol-7; cyan = Pol-8; blue = Pol-9; yellow = Pol-10.

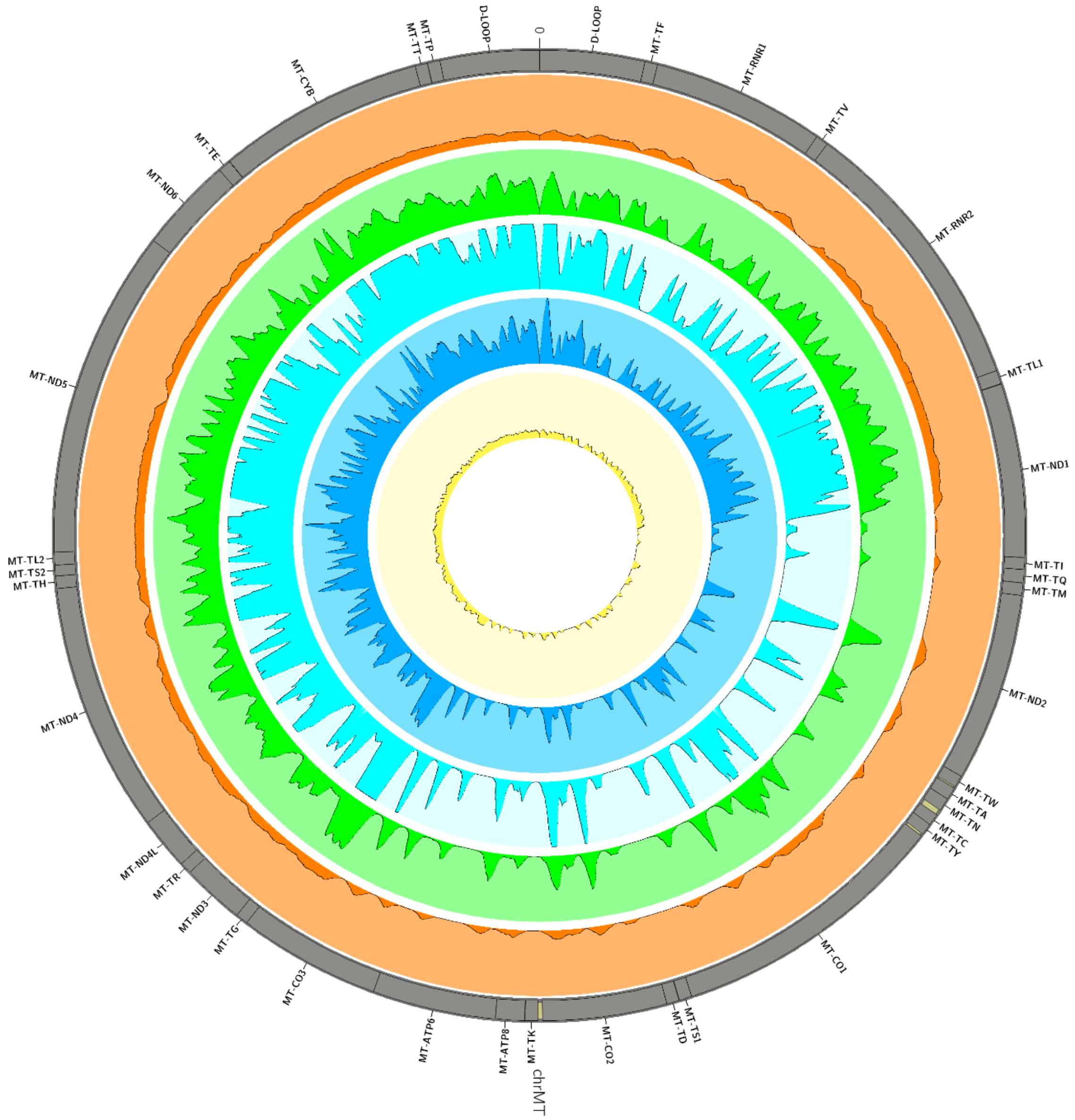

Figure S3. Mean mtDNA depth of coverage. The mean per base depth of coverage was calculated for Polizzello samples. Minimum depth in each histogram = 0, maximum depth = 1000. Color legend for histograms: orange = Pol-11; green = Pol-12; cyan = Pol-13; blue = Pol-14; yellow = Pol-15.

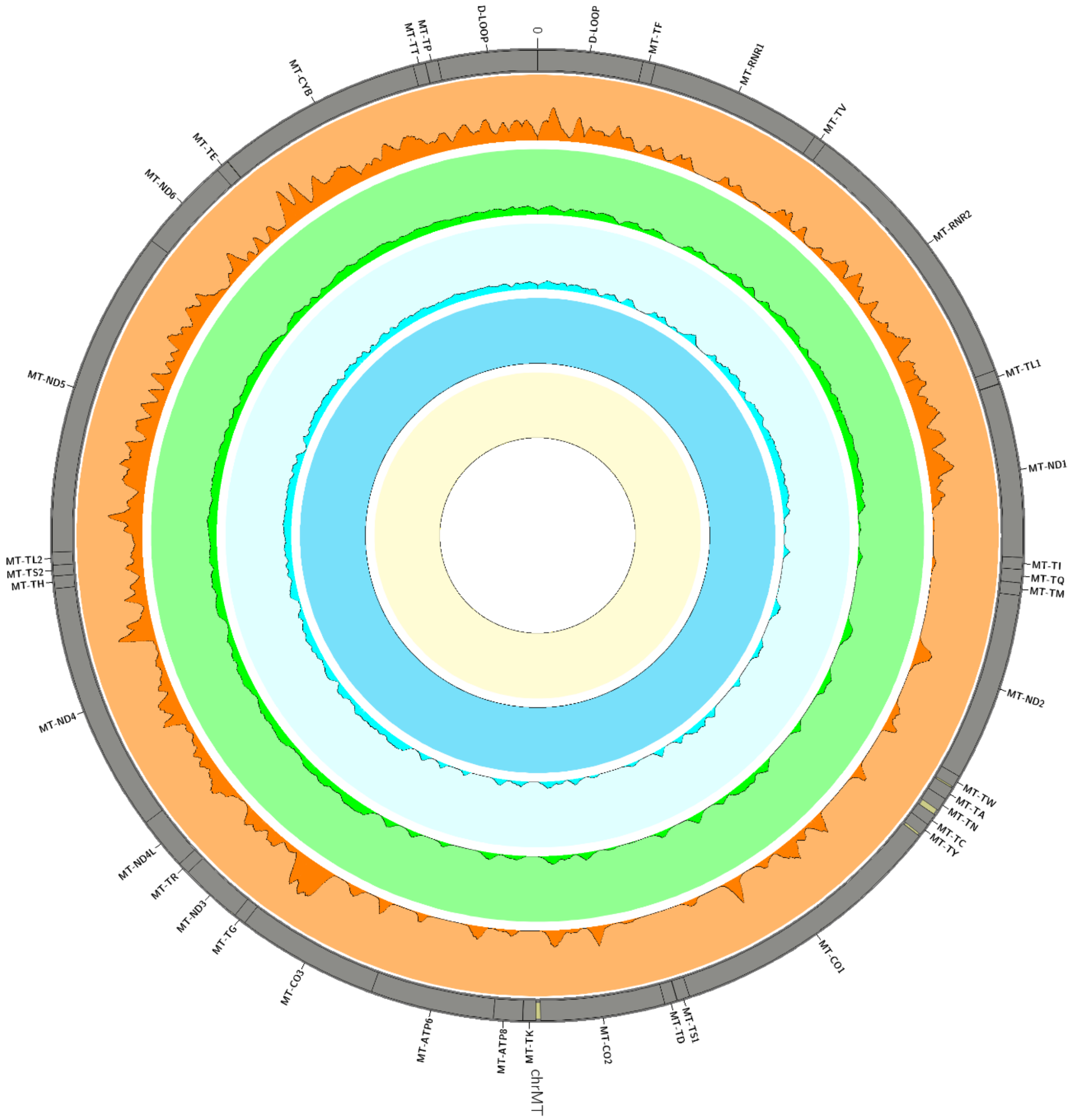

Figure S4. Mean mtDNA depth of coverage. The mean per base depth of coverage was calculated for Polizzello samples. Minimum depth in each histogram = 0, maximum depth = 1000. Color legend for histograms: orange = Pol-16; green = Pol-17; cyan = Pol-18; blue = Pol-19; yellow = Pol-20.

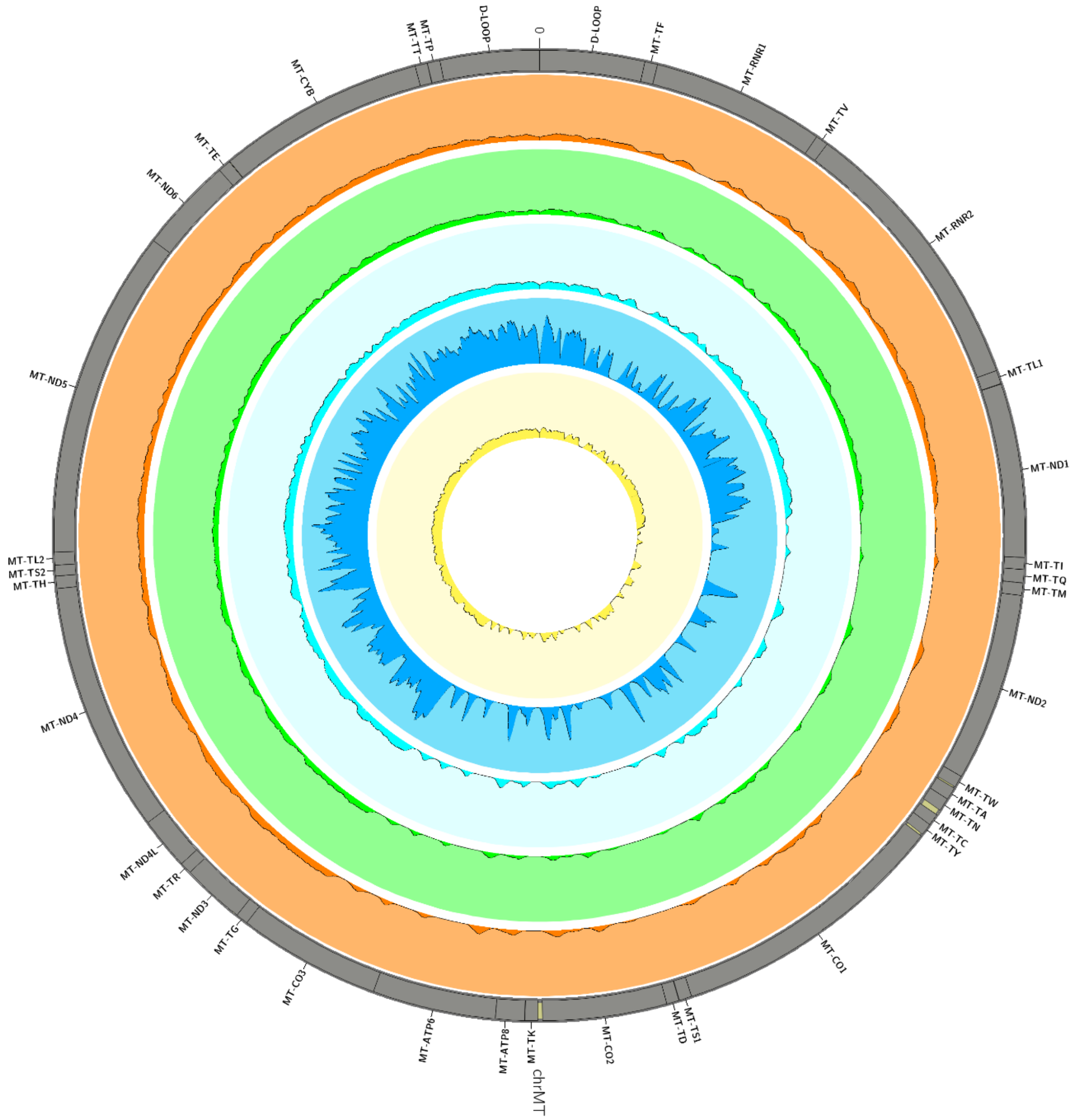

Figure S5. Mean mtDNA depth of coverage. The mean per base depth of coverage was calculated for Polizzello samples. Minimum depth in each histogram = 0, maximum depth = 1000. Color legend for histograms: orange = Pol-21; green = Pol-22; cyan = Pol-23; blue = Pol-24; yellow = Pol-25.

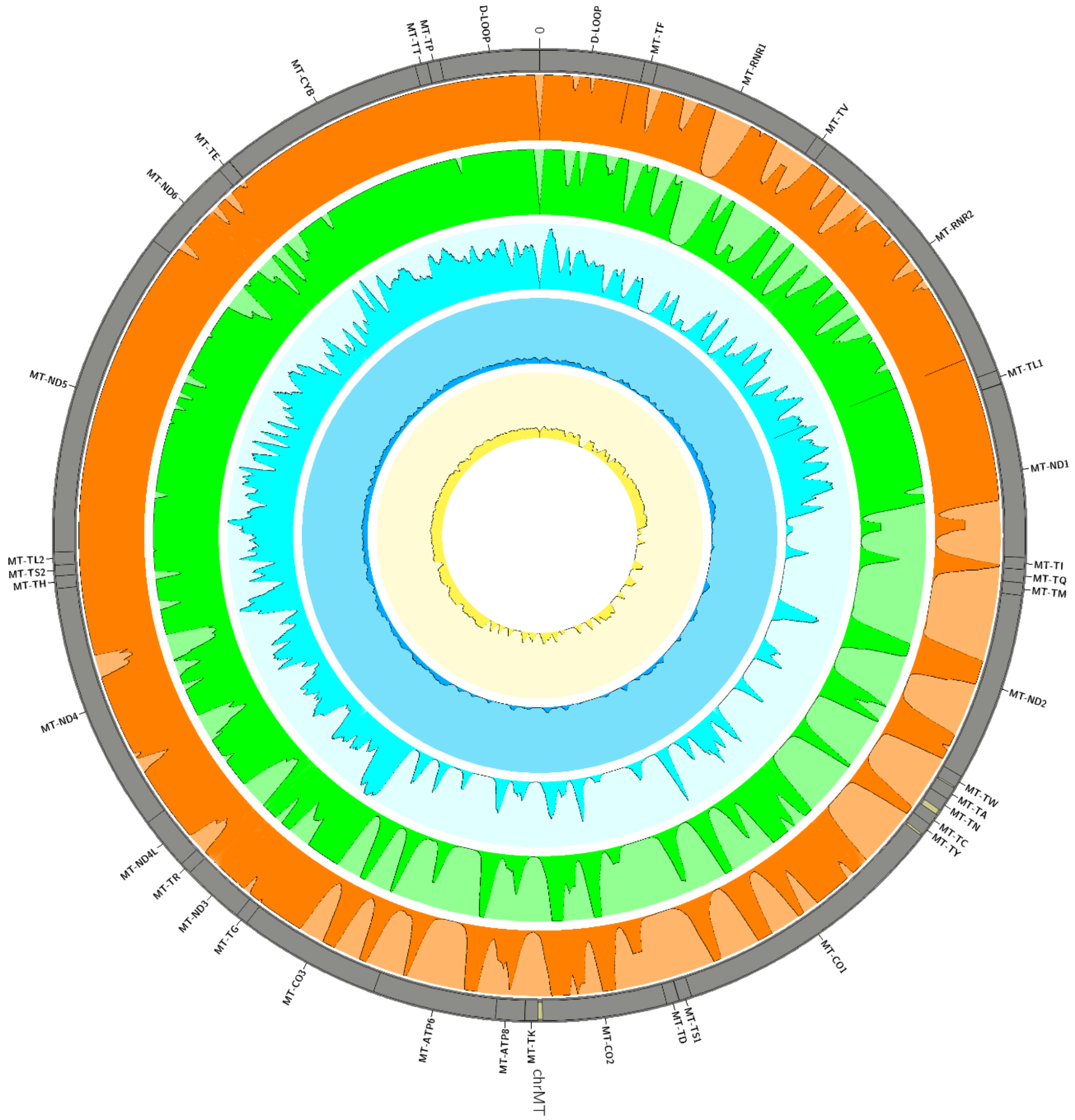

Figure S6. Mean mtDNA depth of coverage. The mean per base depth of coverage was calculated for Polizzello samples. Minimum depth in each histogram = 0, maximum depth = 1000. Color legend for histograms: orange = Pol-26; green = Pol-27; cyan = Pol-28; blue = Pol-29; yellow = Pol-30.

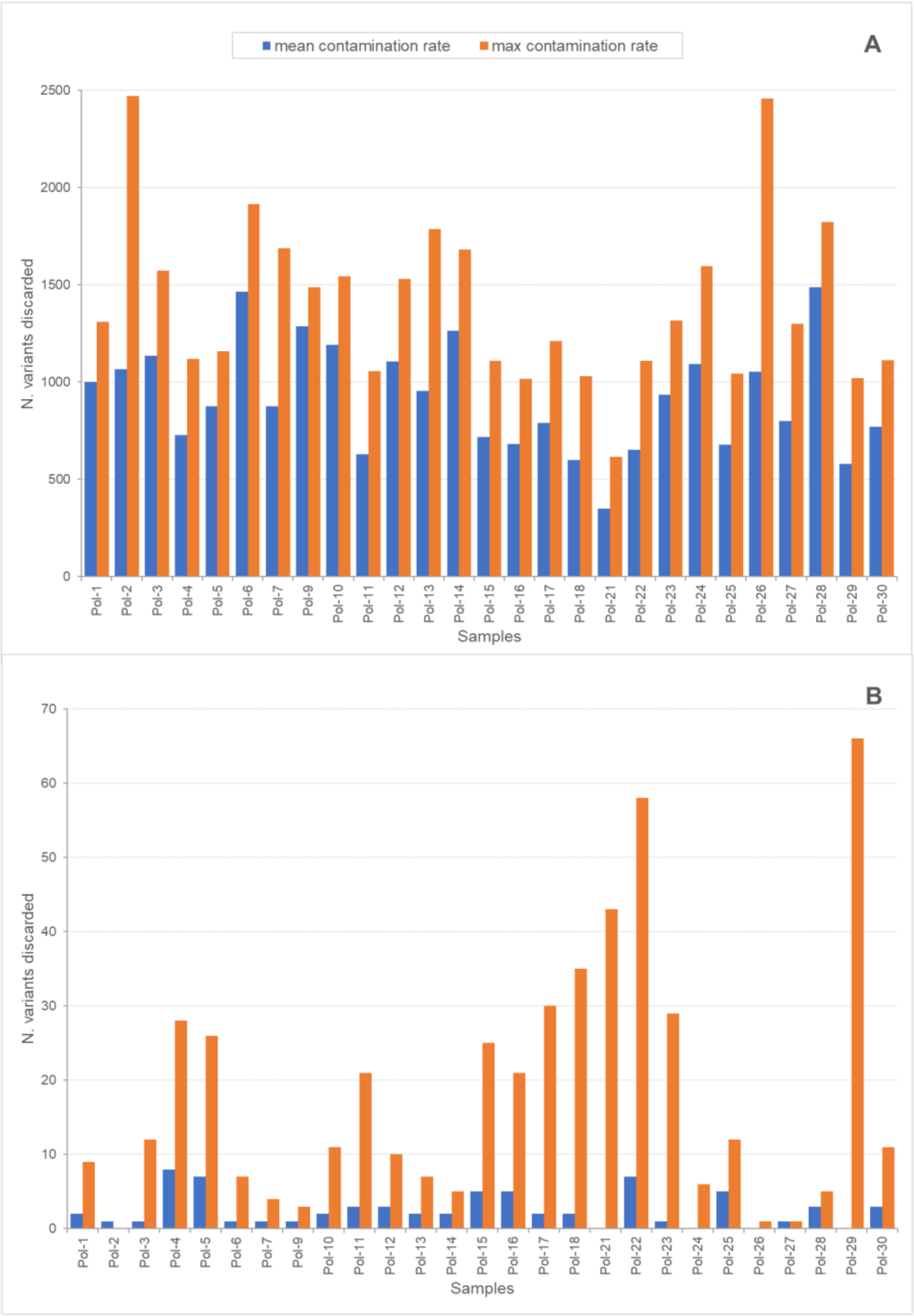

Figure S7. Effect of the contamination rate (CR) parameter on GATK Mutect2 variant filtering process. Mean and highest CR values were previously computed by schmutzi. (A) number of variants filtered out in each sample when considering contamination as one of the reasons; (B) number of variants discarded in each sample exclusively due to contamination.

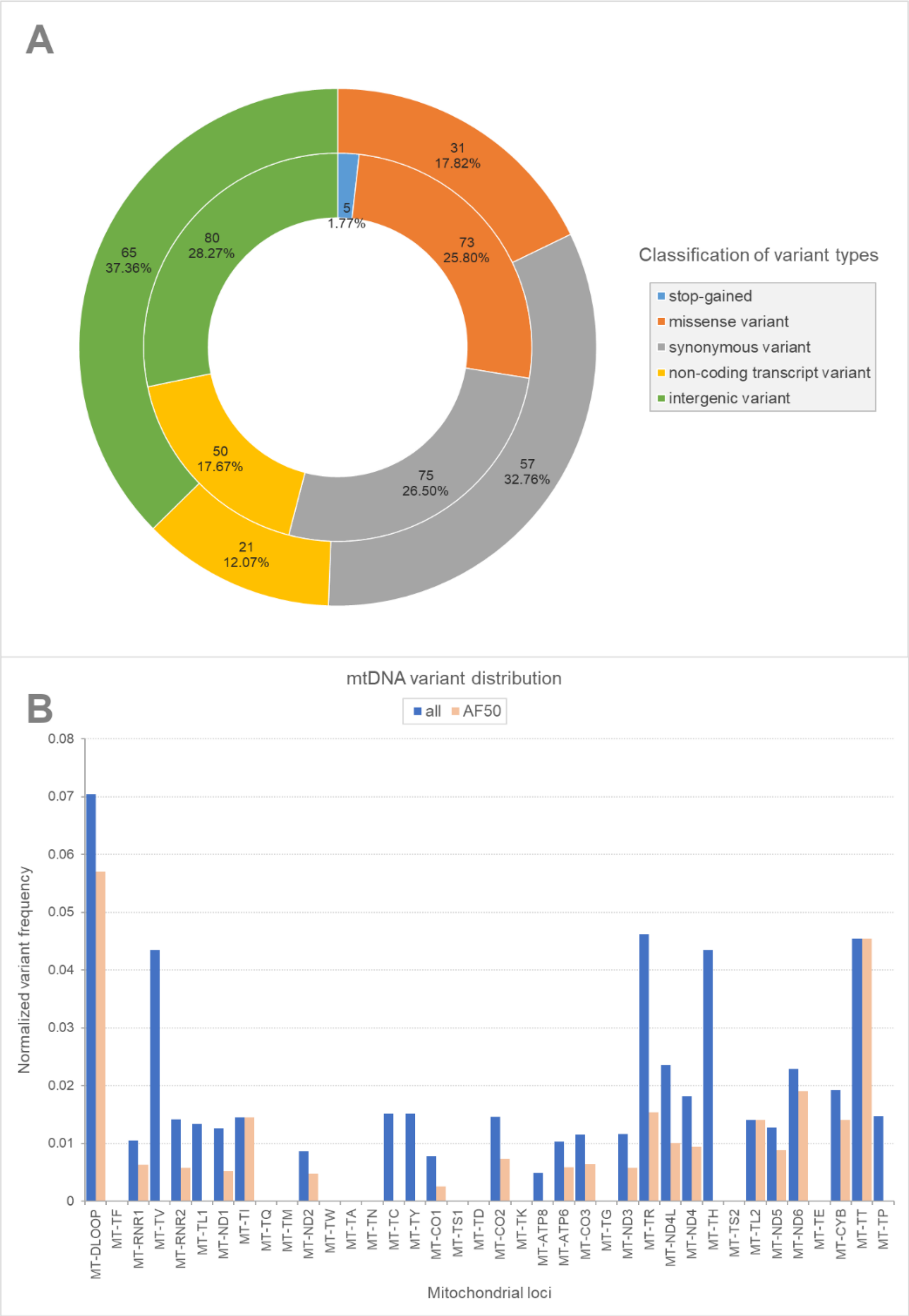

Figure S8. Classification and distribution of variants in Polizzello samples. (A) In the inner circle, variants detected in the whole dataset filtered by fixing minimum allele depth=10 and contamination rate=0.02 were considered, while in the outer circle the classification regards the subset of variants with AF>= 50%. Multiple bases substitutions (MNPs) were not included. (B) Variant frequency normalization was obtained by the ratio between the number of mtDNA variants and the length of mtDNA loci.
